# Effects of *Microbacterium algeriense* C14 on growth and rhizosphere environment of *Zinnia elegans* under cadmium and nickel stress

**DOI:** 10.3389/fmicb.2026.1831418

**Published:** 2026-06-04

**Authors:** Hansheng Liu, Shengxu Wang, Jie Wang, Xingyu Ma, Chunli Zhao, Mingtang Li

**Affiliations:** 1 College of Resources and Environment, Jilin Agricultural University, Changchun, China; 2 College of Forestry and Grassland Science, Jilin Agricultural University, Changchun, China; 3 College of Engineering and Technology, Jilin Agricultural University, Changchun, China

**Keywords:** cadmium-nickel co-contamination, *Microbacterium algeriense*, physiological regulation, plant–microbe combined remediation, remediation mechanism, *Zinnia elegans*

## Abstract

**Introduction:**

Although plantmicrobe systems have been studied for singlemetal contamination, little is known about their synergistic mechanisms under CdNi costress. Current studies often lack mechanistic explanations of microbial immobilization and plant physiological regulation, which this study aims to address.

**Methods:**

To explore the remediation effect and intrinsic mechanism of the plant-microbe combined system on cadmium (Cd)-nickel (Ni) co-contaminated soil, *Microbacterium algeriense* (*M. algeriense*) C14 and *Zinnia elegans* (*Z. elegans*) were selected as the research objects. Through indoor culture and pot experiments, we investigated the CdNi resistance, mineralization mechanism, and effects of strain C14 on seed germination, seedling growth, mature plant growth, physiological metabolism, and heavy metal enrichment of *Z. elegans* under CdNi costress.

**Results:**

*M. algeriense* C14 exhibited strong resistance to Ni^2+^ (within 800 mg·L^−1^) and Cd^2+^ (within 120 mg·L^−1^), and could form stable carboxylate precipitates through the coordination of carboxyl groups in amino acids of its metabolites with heavy metal ions. Inoculation with strain C14 significantly alleviated the inhibitory effect of Cd-Ni co-stress on *Z. elegans*, improved seed germination indexes, promoted the growth of seedlings and mature plants, increased the soluble protein content, superoxide dismutase (SOD) and peroxidase (POD) activities of plants, and reduced Cd and Ni enrichment in plants. Correlation and PCA analyses confirmed the coordinated regulation of growth, physiology, and heavy metal uptake by the strain.The core mechanism of their synergistic remediation was the comprehensive effect of heavy metal immobilization by the strain, plant growth promotion, and synergistic regulation of plant heavy metal uptake, enrichment and physiological metabolism.

**Discussion:**

This study confirms that the *M. algeriense* C14 and *Z. elegans* system is a promising phytomicrobial remediation model. Unlike conventional biosorption or microbially induced carbonate precipitation, strain C14 achieves stable heavy metal immobilization via carboxylate‑mediated mineralization, which differs fundamentally from known mechanisms and offers superior long‑term stability. Meanwhile, *M. algeriense* C14 shows exceptional dual resistance to Cd and Ni, strong IAA‑producing and growth‑promoting traits, distinguishing it from most previously reported PGPR.

## 1 Introduction

With the intensification of industrial development and agricultural activities, heavy metal pollution by Cd and Ni has become a major issue in soil environments, posing serious threats to ecological security and safe production of crops (Gao et al., 2026; Mkhonza et al., 2026; Hayyat et al., 2020). Cd and Ni are highly toxic, non-degradable, and bioaccumulative, which can endanger human health through the food chain (Suhani et al., 2021; Jackson and Alloway, 2017; Hussain and Kabir, 2026). Long-term exposure to these metals can cause severe health problems including kidney damage, bone demineralization, neurological disorders, and carcinogenesis (Chețan et al., 2025). These toxic metals primarily originate from anthropogenic activities including mining operations, metal smelting, electroplating industries, application of contaminated fertilizers and pesticides, and improper disposal of industrial waste (Wan et al., 2024; Uddin et al., 2024). Global soil surveys have revealed that Cd and Ni contamination in agricultural soils has reached alarming levels, with concentrations frequently exceeding regulatory thresholds in major agricultural regions, particularly in areas with intensive industrial activities (Hou et al., 2025). Traditional remediation technologies such as soil excavation, chemical stabilization, and soil washing, although effective in certain scenarios, often suffer from limitations including high costs, secondary pollution, soil structure destruction, and long-term sustainability issues (Iqbal et al., 2024; Zhang et al., 2024). In contrast, microbe-plant synergistic remediation technology has emerged as a research hotspot for heavy metal-contaminated soil remediation due to its advantages of low cost, environmental friendliness, long-lasting remediation effect, and potential for simultaneous restoration of soil ecological functions (Umaru and Owuama, 2018; Supreeth, 2022; Patel et al., 2024; Chojnacka et al., 2023; Kaur and Saxena, 2024; Komire et al., 2024). This biotechnology-based approach not only facilitates the removal or immobilization of toxic metals but also enhances soil fertility and microbial diversity, making it an attractive option for sustainable land management (Kaushal and Pati, 2025).

Plant growth-promoting rhizobacteria (PGPR) can alleviate the damage of heavy metal stress to plants and improve plant adaptability to polluted environments through multiple pathways, such as producing plant hormones, activating nutrients, secreting chelating agents, and inducing plant antioxidant systems (Oleńska et al., 2020; de Andrade et al., 2023). Key mechanisms include: (1) production of 1-aminocyclopropane-1-carboxylate (ACC) deaminase, which reduces ethylene levels in plants under stress conditions and promotes root development (Qin et al., 2024; Alonazi et al., 2025); (2) synthesis of siderophores that enhance iron bioavailability while simultaneously chelating toxic metals, thereby reducing their phytotoxicity (Kaushal and Pati, 2025); (3) secretion of organic acids and exopolysaccharides that alter metal speciation and bioavailability in the rhizosphere (Sahoo et al., 2024); and (4) biofilm formation that facilitates efficient root colonization and metal sequestration (Iqbal et al., 2024). Specific PGPR genera including *Bacillus*, *Pseudomonas*, *Azospirillum*, and *Rhizobium* have demonstrated remarkable efficacy in promoting plant growth while mitigating heavy metal toxicity in contaminated soils (Naveed et al., 2020). Recent studies have shown that multi-strain PGPR consortia often exhibit synergistic effects superior to single-strain inoculations, providing enhanced protection against combined heavy metal stress (Kaushal and Pati, 2025). Screening strains with heavy metal resistance and growth-promoting functions, and clarifying their mechanisms of action, are crucial for achieving synergistic remediation. As a common ornamental plant, *Zinnia elegans* Jacq. has the characteristics of rapid growth, strong adaptability, large biomass production, and extensive fibrous root system, making it particularly suitable for phytoremediation applications (Ahsan et al., 2024; Singh et al., 2024; Mohammadi et al., 2024). The use of ornamental plants for remediation offers additional economic benefits through their esthetic value and potential for commercial cultivation, addressing one of the major limitations of traditional hyperaccumulator plants that often lack economic value (Khan et al., 2021). Recent physiological and molecular studies have revealed that *Z. elegans* possesses efficient metal uptake and translocation mechanisms, coupled with robust antioxidant defense systems that enable it to tolerate elevated metal concentrations in shoots while maintaining normal growth and flowering (Singh et al., 2024; Ahsan et al., 2024). Furthermore, the plant’s tolerance to various abiotic stresses, including salinity and osmotic stress, suggests underlying molecular mechanisms that may be harnessed for improved phytoremediation performance (Mohammadi et al., 2024). However, these studies predominantly address single-metal contamination, leaving a critical gap in understanding Cd-Ni co-stress responses. Furthermore, ornamental plants like *Z. elegans* have received little attention in this context, and the mechanistic links between PGPR colonization, plant physiology, and metal immobilization under dual-metal stress remain unclear.

Although previous studies have focused on single-metal contamination or traditional hyperaccumulator plants, little is known about the synergistic mechanisms of PGPR and ornamental plants under Cd-Ni co-stress. To address this gap, this study isolated a multifunctional PGPR strain C14 was isolated and screened from the rhizosphere soil of *Z. elegans* in the Cd-Ni contaminated area of Hongqiling Town, and identified as *M. algeriense*. By systematically studying the Cd-Ni resistance and mineralization mechanism of this strain, as well as its effects on the growth, development and physiological metabolism of *Z. elegans* under Cd-Ni co-stress and the intrinsic mechanisms, this study aims to provide theoretical support and technical reference for the microbe-plant synergistic remediation of Cd-Ni contaminated soil.

## 2 Materials and methods

### 2.1 Experimental materials

#### 2.1.1 Strain source

Strain C14 was isolated and screened from the rhizosphere soil of Asteraceae plants collected from 6 sampling sites around nickel factories and along major roads in Hongqiling Town, Jilin Province. The sampling areas included farmer greenhouses, farmland streams, agricultural fields, riverbanks of the town, urban green spaces, and nickel factories. A total of 18 types of rhizosphere soil of Asteraceae plants were collected. After isolation, purification, Cd-Ni resistance screening, and functional identification, strain C14 was determined as the target strain.

#### 2.1.2 Test plant

Seeds of *Z. elegans* with a thousand-grain weight of 9.5473 g were purchased from a local flower market and surface-sterilized before sowing.

#### 2.1.3 Experimental reagents and instruments

LB medium, Ashby solid medium, PKO medium, potassium-solubilizing medium, etc.; analytical grade reagents such as CdCl_2_·2.5H_2_O and NiCl_2_·6H_2_O; reagents for determining physiological indicators including Coomassie Brilliant Blue G-250, thiobarbituric acid, and guaiacol. Main instruments included: constant temperature shaking incubator, artificial climate chamber, atomic absorption spectrophotometer, X-ray photoelectron spectrometer (XPS), X-ray diffractometer (XRD), microplate reader, WinRHIZO 2016 root measurement and analysis software, and high-throughput sequencing platform.

### 2.2 Experimental design

#### 2.2.1 Identification and functional characterization of strain C14

The taxonomic status of the strain was determined by 16S rRNA gene sequencing using the universal bacterial primer pair 27F (5′-AGAGTTTGATCMTGGCTCAG-3′)/1492R (5′-TACGGYTACCTTGTTACGACTT-3′). The PCR amplification products were detected by 1% agarose gel electrophoresis with DL2000 DNA Marker (Takara, Japan) as the molecular weight standard, to verify the size and specificity of the amplified 16S rRNA gene fragments. The target bands were excised and purified using a gel extraction kit, and then subjected to Sanger sequencing with the BigDye^®^ Terminator v3.1 Cycle Sequencing Kit (Applied Biosystems, United States) on an ABI 3730xl DNA Analyzer. Raw sequences were assembled and corrected, and homologous sequences were searched using the BLAST program in the NCBI GenBank database. Multiple sequence alignment was performed using ClustalW, and a neighbor-joining (NJ) phylogenetic tree was constructed using MEGA 11 software with 1,000 bootstrap replications to assess the confidence of the tree topology, which was used for the taxonomic identification of the strain.

The obtained sequences were assembled and analyzed for taxonomic identification. The growth curves of the strain under different concentrations of Ni^2+^, Cd^2+^, and pH conditions were measured by spectrophotometry to analyze its resistance and adaptability. The indole-3-acetic acid (IAA) production capacity of the strain was determined by the Salkowski colorimetric method. XPS and XRD techniques were used to analyze the mineralization products and mechanisms of the strain for Cd^2+^ and Ni^2+^. The main components of the strain responsible for Cd-Ni removal were identified through heavy metal removal experiments with different fermentation broth fractions.

#### 2.2.2 Effects of strain C14 on seed germination and seedling growth of *Zinnia elegans* under Cd-Ni stress

Cd stress concentrations were set as follows: 0 mg·L^−1^ (CK), 0.3 mg·L^−1^ (C1), 30 mg·L^−1^ (C2), 120 mg·L^−1^ (C3); Ni stress concentrations were: 0 mg·L^−1^ (CK), 50 mg·L^−1^ (N1), 200 mg·L^−1^ (N2), 600 mg·L^−1^ (N3); combined Cd-Ni stress concentrations included 4 levels of Cd^2+^ (0, 0.3, 30, 120 mg·L^−1^) and 4 levels of Ni^2+^ (0, 50, 200, 600 mg·L^−1^), resulting in 10 combined treatments. The above concentrations were selected based on relevant national environmental standards and preliminary toxicity tests to ensure environmental relevance and toxicity representativeness. For each treatment, two subgroups were set: with strain C14 inoculation (bacterial suspension OD_600_ = 0.8, addition amount 0.5 mL/Petri dish) and without strain inoculation, with three replicates per treatment (Table 1).

**Table 1:** Addition of *Microbacterium algeriense* C_14_ in combination with *Z. elegans* under compound stress of cadmium-nickel at different mass concentrations.

*Microbacterium algeriense* C_14_		Ni Stress Concentration (mg·L^−1^)			
	−/+	0	50	200	600
Cd stress concentration (mg·L^−1^)	0	CK/CKJ	–	–	–
	0.3	–	C1N1/C1N1J	C1N2/C1N2J	C1N3/C1N3J
	30	–	C2N1/C2N1J	C2N2/C2N2J	C2N3/C2N3J
	120	–	C3N1/C3N1J	C3N2/C3N2J	C3N3/C3N3J

The Petri dish filter paper method was used. Thirty plump seeds were placed in each Petri dish, 10 mL of heavy metal solution with corresponding concentration was added, and the Petri dishes were incubated in an artificial climate chamber (temperature 19 ± 4 °C, relative humidity 50–90%, 24 h dark treatment followed by 7 days of culture). Seed germination potential, germination rate, germination index, and vigor index were determined; seedling stem length, root length, root surface area, root projected area, and root volume were measured using WinRHIZO 2016 software.

#### 2.2.3 Effects of strain C14 on growth and physiological metabolism of mature *Zinnia elegans* plants under Cd-Ni stress

Pot experiments were conducted. Soil was mixed with garden soil and sand at a ratio of 3:1, and different concentrations of heavy metal solutions were added for passivation for 30 days before use. *Z. elegans* seedlings with 2–4 true leaves were transplanted into flower pots (1.5 kg soil per pot). For the strain C14 inoculation group, 5 mL of bacterial suspension (OD_600_ = 0.8) was applied to the root zone at the time of transplanting and 25 days after transplanting, with 3 seedlings per pot. After 50 days of conventional management, indicators were determined.

Growth indicators: plant height, stem width, aboveground fresh weight, underground fresh weight, root-shoot ratio; physiological indicators: soluble protein content (Coomassie Brilliant Blue G-250 staining method), MDA content (thiobarbituric acid method), SOD activity (NBT photoreduction method), POD activity (guaiacol method); enrichment indicators: Cd and Ni contents in aboveground and underground parts of plants were determined by atomic absorption spectrophotometer.

#### 2.2.4 Correlation analysis of plant indicators

Correlation analysis was used to explore the correlation among physiological indicators, growth indicators and heavy metal content of *Z. elegans* at the seedling and mature stages under Cd-Ni co-stress, so as to clarify the intrinsic relationship between various indicators.

#### 2.2.5 Principal component analysis of plant indicators

Principal component analysis (PCA) was performed using R software (version 4.4.0) with the vegan package to evaluate multivariate relationships among growth, physiological, and heavy metal indicators. All data were standardized before analysis. PCA score plots were visualized using the ggplot2 package, and the variance interpretation rate of the first two principal components was recorded.

### 2.3 Data processing

SPSS 20.0 software was used for significance analysis by Duncan’s new multiple range test; MEGA 11.0 was used to construct the strain phylogenetic tree; R language (4.4.0) psych package was used for Spearman correlation analysis, and corrplot package was used for heatmap visualization; principal component analysis (PCA) was performed using the vegan package, and PCA plots were visualized with the ggplot2 package; graphs were created using Word and Origin software.

## 3 Results and analysis

### 3.1 Identification and functional characteristics of strain C14

#### 3.1.1 Strain identification

**Figure 1:**
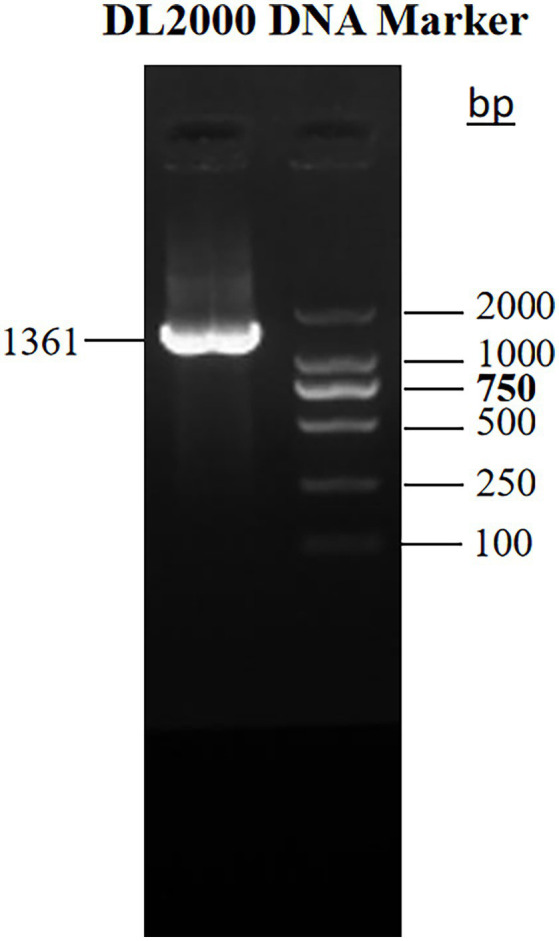
Agarose gel electrophoresis of the 16S rRNA gene PCR amplification product.

**Figure 2:**
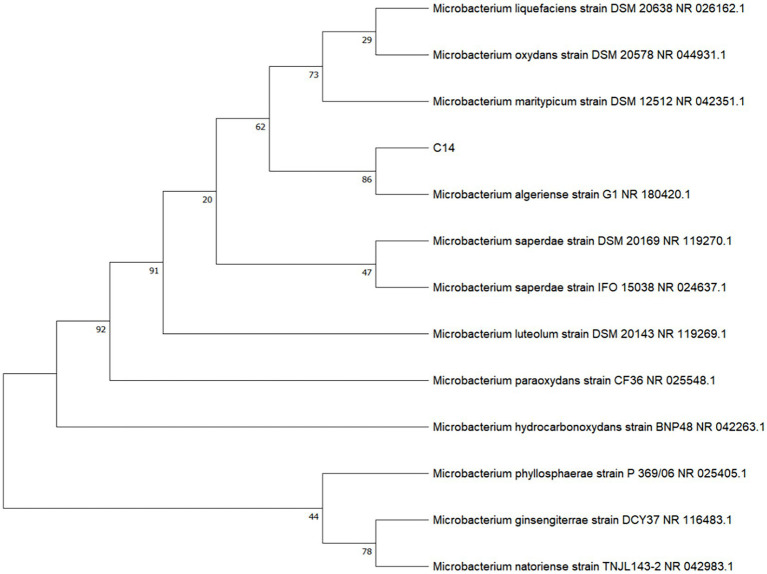
Phylogenetic tree of strain C14 based on 16S rRNA gene sequence. This figure is also presented in another manuscript by the authors.

**Figure 3:**
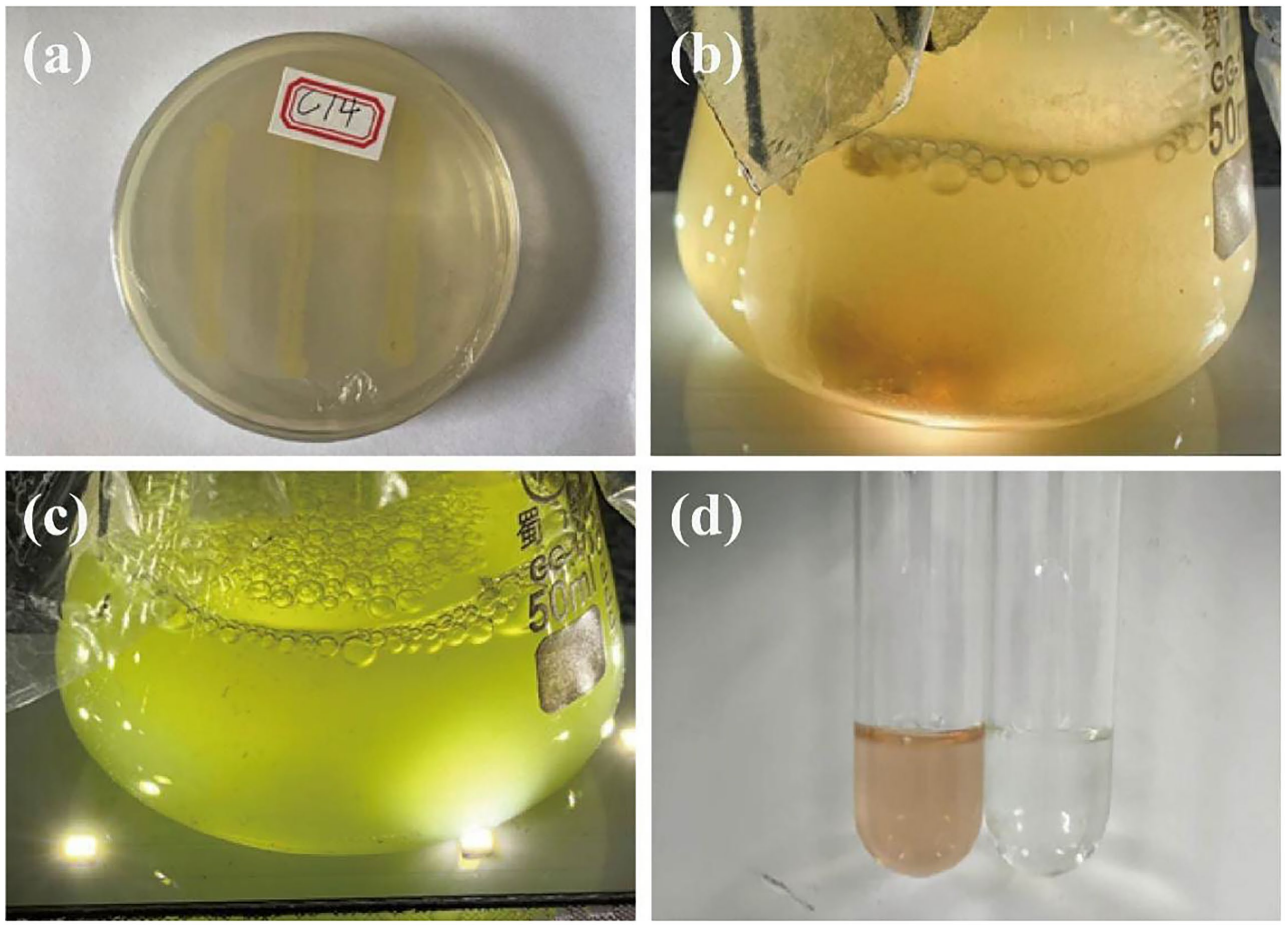
Verification diagram of colony morphology and function of strain C14: **(a)** Colony morphology, **(b)** Cd precipitation product, **(c)** Ni precipitation product, **(d)** IAA assay. This figure is also presented in another manuscript by the authors.

As shown in Figure 1, agarose gel electrophoresis with DL2000 DNA Marker revealed a clear and specific target band of approximately 1,361 bp, which was consistent with the expected size of the amplified fragment, confirming the reliability of the PCR amplification. The purified PCR product was sequenced with the BigDye^®^ Terminator v3.1 kit, and the 16S rRNA gene sequence was used for strain identification.16S rRNA gene sequence analysis showed that strain C14 had a similarity of nearly 100% with *Microbacterium algeriense* (Genbank: NR 180420.1). To further confirm its taxonomic status, a phylogenetic tree was constructed based on the 16S rRNA sequence of strain C14 and other similar type strains (Figure 2). The results showed that strain C14 clustered with the type strain of *M. algeriense* with high confidence, which further verified the taxonomic status of the strain. Combined with colony morphological characteristics (yellowish-white, neat edges, smooth surface, convex in the middle), it was identified as *M. algeriense* or its subtype. The colony morphology and core function verification results of strain C14 are shown in Figure 3.

#### 3.1.2 Heavy metal resistance

**Figure 4:**
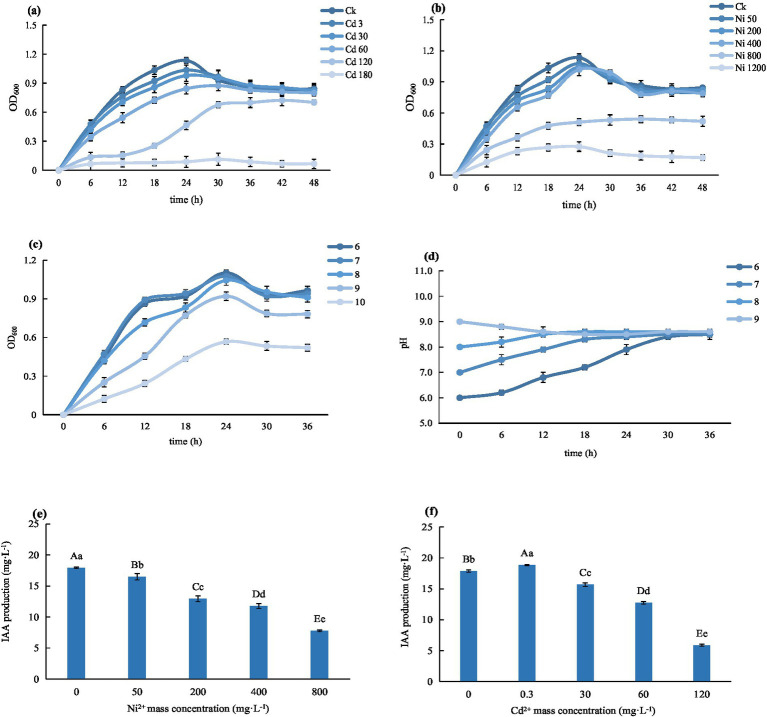
Effects of different concentrations of Ni^2+^, Cd^2+^, and medium pH on the growth and IAA production of strain C14. **(a)** Effects of different concentrations of Cd^2+^ on strain growth, **(b)** Effects of different concentrations of Ni^2+^ on strain growth, **(c)** Effects of medium pH on strain growth, **(d)** Effects of medium pH on fermentation broth pH, **(e)** Effects of different concentrations of Ni^2+^ on IAA production of strain C14, **(f)** Effects of different concentrations of Cd^2+^ on IAA production of strain C14. This figure is also presented in another manuscript by the authors.

Strain C14 could maintain certain cell activity at Ni^2+^ concentrations up to 800 mg·L^−1^ and Cd^2+^ concentrations up to 120 mg·L^−1^; the logarithmic growth phase was 0–6 h, and the growth peak was reached at 24 h (OD_600_ values were 1.134 and 1.137, respectively); when Ni^2+^ concentration reached 1,200 mg·L^−1^ and Cd^2+^ concentration reached 180 mg·L^−1^, the growth of the strain was significantly inhibited. The effects of different concentrations of Ni^2+^ and Cd^2+^ on the growth of strain C14 are shown in Figures 4a,b. The higher the heavy metal concentration and the longer the culture time, the higher the mortality rate of the strain. The mortality rate reached 98.67% after 12 h of treatment with 2,400 mg·L^−1^ Ni^2+^.

#### 3.1.3 Environmental adaptability

As shown in Figure 4c, strain C14 could grow normally at pH 6–9, reaching the maximum growth at 24 h; growth was significantly slowed at pH 10. The strain could adjust the pH of the fermentation broth to an alkaline environment suitable for growth through its own regulation (Figure 4d). When the initial pH was 6–8, the pH of the fermentation broth increased slowly; when the initial pH was 9–10, it decreased slowly, and stabilized after 30–36 h.

#### 3.1.4 Growth-promoting ability and mineralization mechanism

Strain C14 had the ability to produce IAA. Low concentrations of Cd^2+^ promoted IAA production, while high concentrations of Cd^2+^ and Ni^2+^ inhibited IAA production. However, a certain amount of IAA could still be produced under the stress of 800 mg·L^−1^ Ni^2+^ and 120 mg·L^−1^ Cd^2+^. The effects of different concentrations of Ni^2+^ and Cd^2+^ on the IAA production of strain C14 are shown in Figures 4e,f, respectively.

**Figure 5:**
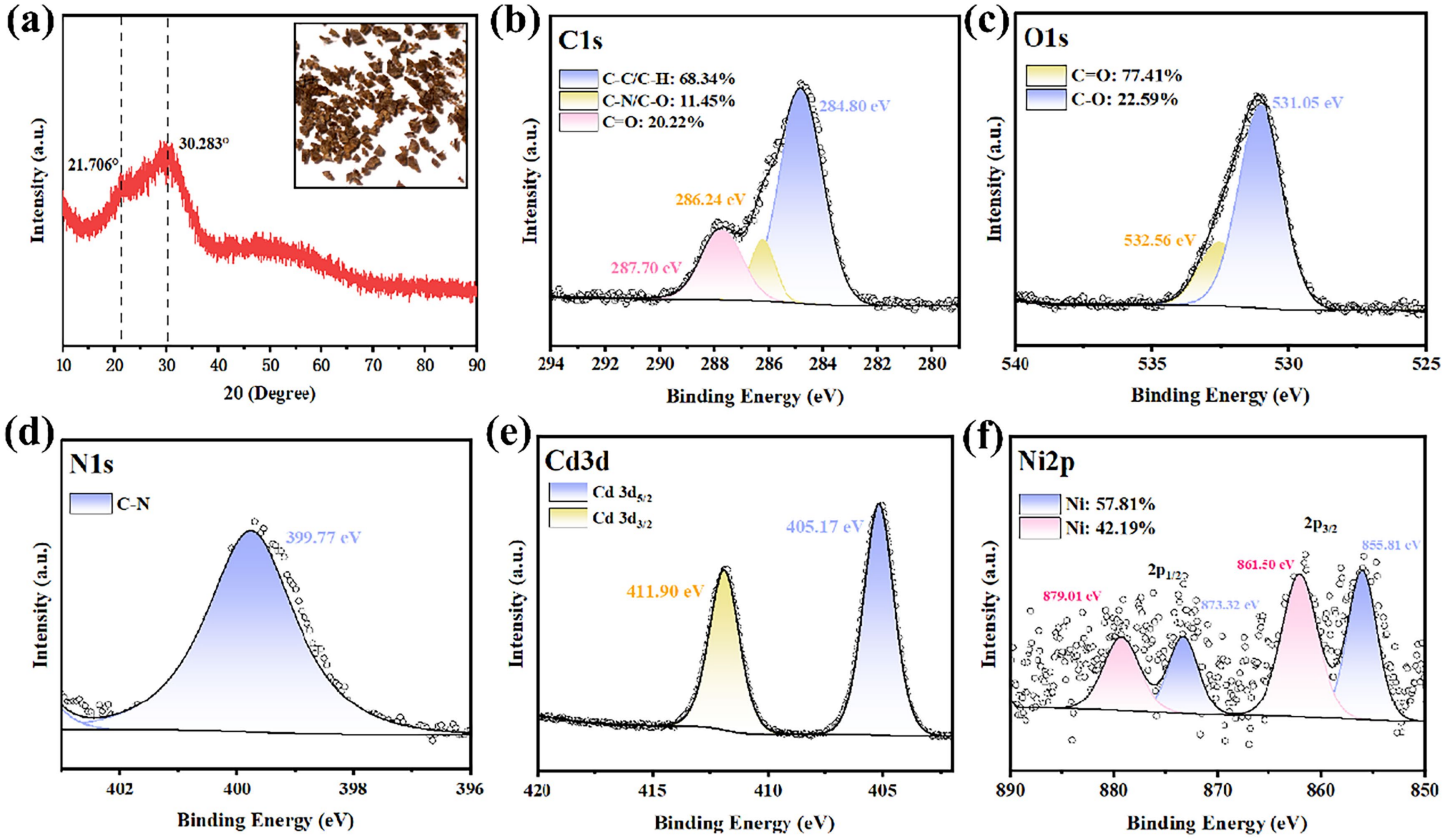
Characterization of coordination precipitation products between metabolites of strain C14 and Cd^2+^, Ni^2+^. **(a)** XRD spectrum of Cd-Ni mineralization product, with an inset showing the digital photograph of the mineralization product. XPS spectra of Cd-Ni mineralization product: **(b)** C1s spectrum, **(c)** O1s spectrum, **(d)** N1s spectrum, **(e)** Cd3d spectrum, **(f)** Ni2p spectrum.

As shown in Figure 5, XPS and XRD analyses confirmed that carboxyl groups in amino acid metabolites act as active sites to coordinate with Cd^2+^ and Ni^2+^, forming stable amorphous carboxylate precipitates. This carboxylate-mediated mineralization is mechanistically distinct from typical biosorption, bioaccumulation, or carbonate/phosphate precipitation, and provides more stable immobilization with lower metal re-mobilization risk.

### 3.2 Effects of strain C14 on seed germination and seedling growth of *Zinnia elegans* under Cd-Ni stress

#### 3.2.1 Seed germination indicators

Cd-Ni co-stress had a significant inhibitory effect on the seed germination and seedling growth of *Z. elegans*, and the higher the stress concentration, the stronger the inhibitory effect. Low-concentration co-stress (C1N1: 0.3 mg·L^−1^ Cd^2+^ + 50 mg·L^−1^ Ni^2+^) had a weak inhibitory effect on seed germination, and even some germination indexes were slightly improved. However, under high-concentration co-stress (C3N3: 120 mg·L^−1^ Cd^2+^ + 600 mg·L^−1^ Ni^2+^), the germination potential, germination rate and vigor index of *Z. elegans* seeds were significantly decreased by 52.93, 59.01 and 85.17% compared with the control group, respectively. The growth indicators such as seedling stem length, root length and root surface area were all greatly reduced, and the root-shoot ratio was significantly decreased, indicating that the inhibitory effect of heavy metal co-stress on the root growth of *Z. elegans* was more obvious.

Low-concentration Cd-Ni stress (0.3 mg·L^−1^ Cd^2+^, 50 mg·L^−1^ Ni^2+^) had a certain promoting effect on the seed germination of *Z. elegans*, while high concentration significantly inhibited it. Inoculation with strain C14 significantly increased the germination potential, germination rate, germination index and vigor index of seeds under the same stress concentration. Under Cd-Ni co-stress, the C1N1 treatment (0.3 mg·L^−1^ Cd^2+^ + 50 mg·L^−1^ Ni^2+^) had the optimal germination indexes. After inoculation with strain C14, the germination indexes of all co-stress treatments were significantly higher than those of the non-inoculated group, with the germination potential and germination rate increased by 57.14 and 65.28%, respectively, under high-concentration co-stress (C3N3). The effects of inoculating strain C14 on the seed germination indexes of *Z. elegans* under Cd-Ni stress are shown in Table 2.

**Table 2:** Effect of the addition of *M. algeriense* C14 on the germination indicators of *Z. elegans* seeds under compound stress of cadmium and nickel.

Treatment	Germinative force	Germination percentage	Germination index	Vitality index
CK	56.67 ± 3.33BCDEcde	67.78 ± 5.09CDc	7.58 ± 0.93Gj	76.96 ± 7.44De
C1N1	62.22 ± 5.09ABCabc	80.00 ± 3.33ABab	10.92 ± 0.68BCDbcde	92.48 ± 5.15Cc
C1N2	53.33 ± 8.82CDEFdef	74.44 ± 1.92BCb	11.83 ± 0.25ABCab	67.80 ± 4.33Ef
C1N3	47.78 ± 1.92EFGHfg	54.44 ± 1.92EFGf	10.17 ± 0.83CDEdefg	26.30 ± 3.31HIJij
C2N1	48.89 ± 6.94DEFGefg	61.11 ± 3.85DEde	9.44 ± 0.64DEFfgh	48.44 ± 1.75Fg
C2N2	45.56 ± 5.09FGHfgh	52.22 ± 5.09FGfg	9.33 ± 0.88DEFfgh	35.99 ± 7.05Gh
C2N3	37.78 ± 1.92HIJijk	46.67 ± 3.33GHgh	8.89 ± 1.46EFGghi	21.89 ± 4.5IJjk
C3N1	32.22 ± 1.92JKjkl	42.22 ± 5.09HIhij	8.05 ± 0.53FGij	33.29 ± 3.99GHh
C3N2	30.00 ± 3.33JKkl	36.67 ± 3.33IJjk	8.06 ± 0.85FGij	18.79 ± 2.88JKk
C3N3	26.67 ± 3.33Kl	27.78 ± 1.92Kl	7.46 ± 0.47Gj	11.41 ± 1.55Kl
CKJ	64.44 ± 1.92ABab	80.00 ± 3.33ABab	11.58 ± 0.16ABCabc	125.40 ± 2.09Aa
C1N1J	68.89 ± 3.85Aa	83.33 ± 3.33Aa	12.27 ± 0.74ABa	112.24 ± 8.08Bb
C1N2J	58.89 ± 8.39ABCDbcd	78.89 ± 1.92ABab	12.63 ± 0.49Aa	83.89 ± 3.25CDd
C1N3J	51.11 ± 1.92DEFdefg	55.56 ± 3.85EFef	10.97 ± 0.51ABCDbcd	36.68 ± 2.49Gh
C2N1J	53.33 ± 3.33CDEFdef	65.56 ± 1.92Dcd	10.36 ± 1.04CDEcdef	63.34 ± 3.64Ef
C2N2J	50 ± 3.33DEFGefg	53.33 ± 3.33EFGf	10.43 ± 0.38CDEcdef	46.02 ± 1.76Fg
C2N3J	40 ± 3.33GHIJhij	52.22 ± 1.92FGfg	9.75 ± 0.45DEFdefg	30.22 ± 2.64GHIhi
C3N1J	43.33 ± 3.33FGHIghi	43.33 ± 3.33HIhi	9.64 ± 0.41DEFefg	48.05 ± 0.39Fg
C3N2J	33.33 ± 3.33IJKjkl	38.89 ± 5.09HIJij	8.2 ± 0.43FGhij	24.84 ± 1.5HIJijk
C3N3J	32.22 ± 1.92JKjkl	32.22 ± 1.92JKkl	8.25 ± 0.08FGhij	18.43 ± 0.84JKk

Data in the table are mean ± standard deviation; different lowercase letters in the same column indicate significant differences (*P* < 0.05), and different uppercase letters indicate extremely significant differences (*P* < 0.01).

#### 3.2.2 Seedling growth and physiological indicators

As shown in Table 3, Cd-Ni co-stress had a significant inhibitory effect on the seedling growth of *Z. elegans*, and indicators such as stem length, root length and root surface area showed a significant downward trend with the increase of stress concentration. Only the root projected area was slightly increased (by 8.61%) under low-concentration co-stress (C1N1: 0.3 mg·L^−1^ Cd^2+^ + 50 mg·L^−1^ Ni^2+^) compared with the control group. When the concentration of Cd or Ni was fixed alone, the increase of the concentration of the other heavy metal would aggravate the inhibitory effect. For example, compared with the C1N1 treatment, the stem length and root length in the C1N3 treatment (0.3 mg·L^−1^ Cd^2+^ + 600 mg·L^−1^ Ni^2+^) were reduced by 70.09 and 73.67%, respectively. After inoculation with strain C14, the seedling growth indicators under the same stress concentration were significantly improved. The root projected area in the C1N1J treatment was increased by 10.00% compared with the CKJ treatment, and the decrease range of indicators such as stem length and root length at each concentration gradient was smaller than that in the non-inoculated group, which effectively alleviated the inhibitory effect of co-stress.

**Table 3:** Effect of the addition of *M. algeriense* C14 on the growth indicators of *Z. elegans* seeds under compound stress of cadmium and nickel.

Treatment	Stem length	Root length	Root projection area	Root surface area	Root volume
CK	5.252 ± 0.157Bb	4.922 ± 0.382Aa	0.267 ± 0.031CDcd	0.663 ± 0.027ABb	0.011 ± 0.001ABab
C1N1	4.296 ± 0.169Dd	4.179 ± 0.110Bc	0.290 ± 0.006BCbc	0.622 ± 0.010Cc	0.010 ± 0.001ABCbc
C1N2	2.606 ± 0.24GHgh	3.119 ± 0.136De	0.245 ± 0.011DEde	0.464 ± 0.013Ee	0.008 ± 0.001DEFdef
C1N3	1.285 ± 0.092JKl	1.296 ± 0.13HIJlm	0.147 ± 0.015IJhi	0.264 ± 0.011IJi	0.006 ± 0.001FGHgh
C2N1	2.570 ± 0.342GHgh	2.577 ± 0.061Eg	0.211 ± 0.006FGf	0.414 ± 0.008Ff	0.008 ± 0.001CDEFde
C2N2	2.325 ± 0.202Hh	1.510 ± 0.303GHIjkl	0.163 ± 0.015HIgh	0.321 ± 0.022GHh	0.006 ± 0.001FGHgh
C2N3	1.474 ± 0.172IJjkl	0.991 ± 0.174JKno	0.119 ± 0.017Jj	0.234 ± 0.031Jj	0.004 ± 0.001IJKjk
C3N1	2.522 ± 0.16GHh	1.610 ± 0.158GHijk	0.17 ± 0.007HIgh	0.312 ± 0.015Hh	0.006 ± 0.002GHIhi
C3N2	1.496 ± 0.091IJjkl	0.828 ± 0.059KLo	0.074 ± 0.006Kk	0.140 ± 0.006Kkl	0.003 ± 0.001JKLjk
C3N3	0.973 ± 0.109Km	0.555 ± 0.081Lp	0.057 ± 0.005Kk	0.119 ± 0.006Kl	0.001 ± 0.001Ll
CKJ	5.686 ± 0.184Aa	5.14 ± 0.287Aa	0.300 ± 0.028ABb	0.698 ± 0.033Aa	0.012 ± 0.002Aa
C1N1J	4.696 ± 0.151Cc	4.455 ± 0.148Bb	0.330 ± 0.017Aa	0.647 ± 0.015BCbc	0.012 ± 0.001Aab
C1N2J	3.059 ± 0.081EFef	3.584 ± 0.096Cd	0.268 ± 0.009CDcd	0.534 ± 0.011Dd	0.009 ± 0.001BCDcd
C1N3J	1.598 ± 0.121IJijk	1.747 ± 0.096Gij	0.160 ± 0.014HIgh	0.327 ± 0.014GHh	0.007 ± 0.001DEFGefg
C2N1J	3.291 ± 0.24Ee	2.843 ± 0.048DEf	0.232 ± 0.006EFef	0.441 ± 0.005EFef	0.009 ± 0.001BCDEcd
C2N2J	2.603 ± 0.053GHgh	1.810 ± 0.132FGi	0.176 ± 0.008HIg	0.338 ± 0.016GHgh	0.007 ± 0.001EFGefgh
C2N3J	1.672 ± 0.106IJij	1.423 ± 0.043GHIklm	0.145 ± 0.011IJhi	0.274 ± 0.013Ii	0.005 ± 0.001HIJij
C3N1J	2.851 ± 0.115FGfg	2.140 ± 0.095Fh	0.181 ± 0.006GHg	0.360 ± 0.013Gg	0.007 ± 0.001FGHfgh
C3N2J	1.835 ± 0.034Ii	1.195 ± 0.105IJKmn	0.128 ± 0.006Jij	0.250 ± 0.018IJij	0.004 ± 0.001IJKj
C3N3J	1.317 ± 0.083JKkl	0.916 ± 0.028JKLno	0.079 ± 0.005Kk	0.157 ± 0.011Kk	0.002 ± 0.001KLkl

Data in the table are mean ± standard deviation; different lowercase letters in the same column indicate significant differences (*P* < 0.05), and different uppercase letters indicate extremely significant differences (*P* < 0.01).

**Figure 6:**
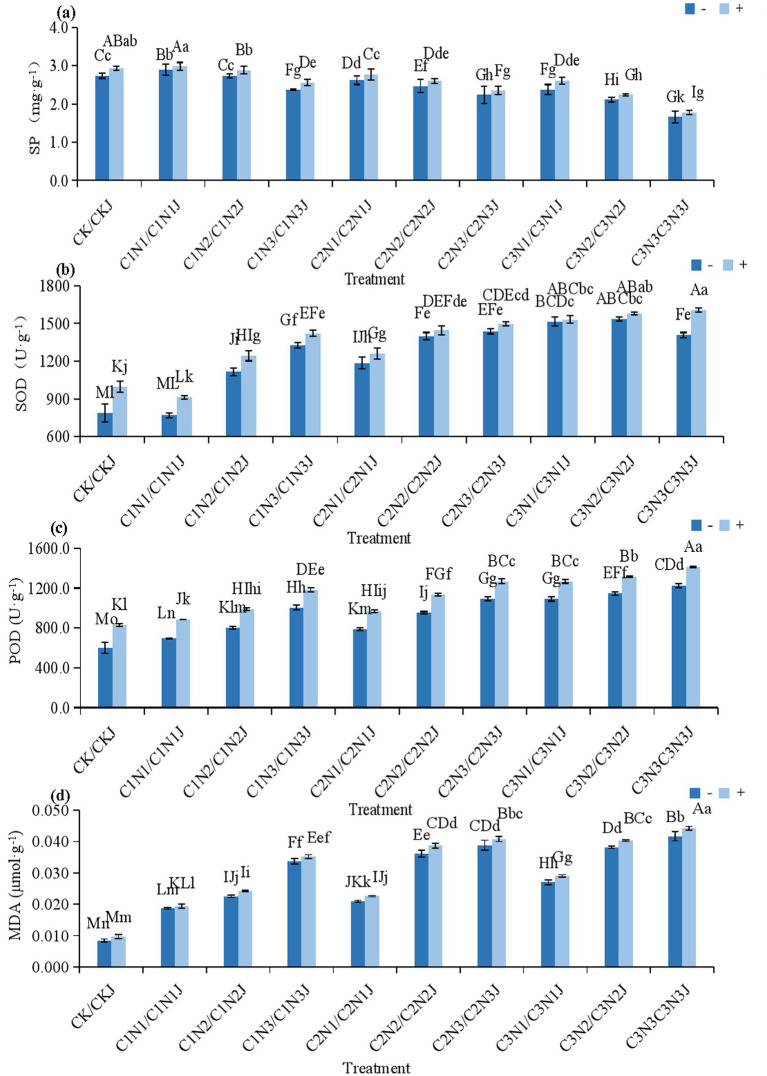
Effects of cadmium and nickel combined stress on physiological indicators of *Z. elegans* Jacq. seedlings added with *M. algeriense* C14: **(a)** Soluble protein, **(b)** superoxide dismutase, **(c)** peroxidase, **(d)** malondialdehyde. In the figure, different lowercase letters in the same figure indicate significant differences (*p* < 0.05), while different uppercase letters in the same figure indicate extremely significant differences (*p* < 0.01). “−” indicates no application, “+” indicates application.

In terms of physiological indicators (Figure 6), without the inoculation of the strain, the soluble protein (SP) content showed a trend of first increasing and then decreasing with the increase of Cd and Ni concentrations, while the activities of SOD, POD and the content of MDA all increased with the increase of stress concentration. The POD activity and MDA content in the C3N3 treatment reached 1225.33 U·g^−1^ and 0.0417 μmol·g^−1^, respectively. After inoculation with strain C14, the SP content and the activities of SOD and POD were significantly increased. The SP content and SOD activity in the C3N3J treatment were increased to 1.78 mg·g^−1^ and 1606.92 U·g^−1^, respectively. Although the MDA content still exhibited an upward trend under heavy metal stress, the inoculated group showed a slightly higher MDA accumulation than the non-inoculated group at the same stress concentration. This unexpected increase may be attributed to inappropriate concentration of IAA produced by the strain, or induced by unknown secondary metabolites produced by the strain, which disrupted the plant’s oxidative balance despite the strain’s potential to alleviate stress.

#### 3.2.3 Enrichment indicators

**Figure 7:**
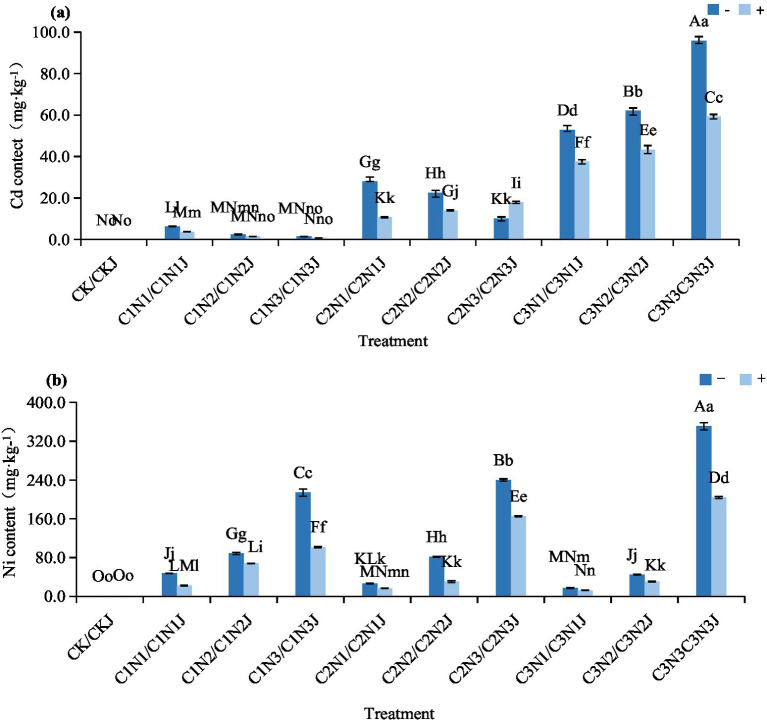
Effects of adding *M. algeriense* C14 on cadmium and nickel content in *Z. elegans* seedlings under combined cadmium and nickel stress **(a)**. Cadmium content **(b)**. Nickel content. In the figure, different lowercase letters in the same figure indicate significant differences (*p* < 0.05), while different uppercase letters in the same figure indicate extremely significant differences (*p* < 0.01). “–” indicates no application, “+” indicates application.

As shown in Figure 7, under Cd-Ni co-stress, the Cd and Ni enrichment characteristics of *Z. elegans* seedlings were significantly affected by the interaction of heavy metal concentrations, and the heavy metal accumulation in plants was effectively regulated after inoculation with strain C14. Under low-concentration Cd stress (0.3 mg·L^−1^), the increase of Ni concentration would inhibit the Cd uptake by seedlings, while when the Cd concentration increased to 30 mg·L^−1^ and above, the promoting effect of Cd itself became dominant, and high-concentration Ni turned into a promoting effect. Without the inoculation of the strain, the Cd content in seedlings reached a peak of 95.8 mg·kg^−1^ in the C3N3 treatment (120 mg·L^−1^ Cd^2+^ + 600 mg·L^−1^ Ni^2+^). When the Ni concentration was fixed, the Cd content increased exponentially with the increase of Cd concentration; and the Cd content in the C3N3J treatment decreased to 59.23 mg·kg^−1^. The inhibitory effect of low-concentration Ni on Cd uptake was more significant.

For Ni enrichment, when the Cd concentration was fixed, the Ni content in seedlings increased significantly with the increase of Ni concentration. Without the inoculation of the strain, the Ni content reached a peak of 350.93 mg·kg^−1^ in the C3N3 treatment. When the Ni concentration was low, the increase of Cd concentration would inhibit Ni uptake, while under high Ni concentration, high Cd concentration promoted Ni accumulation instead; after inoculation with strain C14, the Ni content in seedlings was significantly reduced, with the Ni content in the C3N3J treatment decreasing to 203.61 mg·kg^−1^, and the inhibitory effect of Cd on Ni uptake was enhanced at each Ni concentration gradient. On the whole, the Ni enrichment capacity of *Z. elegans* seedlings was stronger than the Cd enrichment capacity, and the heavy metal content in the underground parts was significantly higher than that in the aboveground parts. Inoculation with strain C14 significantly reduced the Cd and Ni accumulation in seedlings by reducing the content of available heavy metals and regulating the root uptake process of plants, with a better reduction effect on Ni than on Cd, which provided support for alleviating heavy metal toxicity and reducing the risk of the food chain.

### 3.3 Effects of strain C14 on growth and physiological metabolism of mature *Zinnia elegans* plants under Cd-Ni stress

#### 3.3.1 Growth indicators

Cd-Ni co-stress had a significant inhibitory effect on the plant height, stem width, aboveground fresh weight, underground fresh weight and root-shoot ratio of mature *Z. elegans* plants, and the higher the stress concentration, the more obvious the inhibitory effect. Only the plant height, stem width and aboveground fresh weight were slightly increased (by 4.31, 0.88 and 4.02% respectively) under low-concentration co-stress (C1N1: 0.3 mg·L^−1^ Cd^2+^ + 50 mg·L^−1^ Ni^2+^) compared with the control group (CK), and the other indicators all showed a downward trend with the increase of Cd or Ni concentration. For example, the plant height and underground fresh weight in the C3N3 treatment (120 mg·L^−1^ Cd^2+^ + 600 mg·L^−1^ Ni^2+^) were significantly reduced by 65.45 and 87.57% compared with CK, respectively. After inoculation with strain C14, all growth indicators of mature *Z. elegans* plants under the same concentration of co-stress were significantly improved. The plant height, stem width and aboveground fresh weight in the C1N1J treatment (0.3 mg·L^−1^ Cd^2+^ + 50 mg·L^−1^ Ni^2+^ + strain C14) were increased by 5.6, 5.9 and 13.5% compared with the C1N1 treatment, respectively. Although the growth was still inhibited under high-concentration stress, the growth indicators were significantly better than those in the non-inoculated group. For example, the plant height and underground fresh weight in the C3N3J treatment were increased by 44.4 and 56.8% compared with the C3N3 treatment, respectively, which effectively alleviated the inhibitory effect of Cd-Ni co-stress on the growth of mature plants and reflected the promoting effect of strain C14 on the growth of mature *Z. elegans* plants. The effects of inoculating strain C14 on the growth indexes of mature *Z. elegans* plants under Cd-Ni stress are shown in Table 4.

**Table 4:** Effects of cadmium and nickel stress on the growth indicators of *Z. elegans* mature plants under the application of *M. algeriense* C14.

Treatment	Plant height	Stem width	Fresh weight above ground	Underground fresh weight	Root shoot ratio
CK	44.14 ± 2.06Bc	4.53 ± 0.1Bb	2.49 ± 0.04Cd	3.54 ± 0.1Bb	1.42 ± 0.03Aa
C_1_N_1_	46.04 ± 1.46ABbc	4.57 ± 0.03Bb	2.59 ± 0.07BCcd	3.41 ± 0.07Cc	1.31 ± 0.05ABCbc
C_1_N_2_	35.76 ± 1.13CDe	3.51 ± 0.05DEd	2.31 ± 0.03De	2.32 ± 0.08Ff	1.01 ± 0.03Ffg
C_1_N_3_	26.0 ± 1.62FGHgh	2.5 ± 0.04GHf	1.25 ± 0.11GHh	1.08 ± 0.03JKl	0.86 ± 0.05GHhi
C_2_N_1_	35.23 ± 0.34De	3.39 ± 0.04Ed	1.88 ± 0.05Ef	2.22 ± 0.07FGg	1.19 ± 0.02CDde
C_2_N_2_	31.53 ± 0.98Ef	2.57 ± 0.08Gf	1.56 ± 0.1Fg	1.79 ± 0.06Hi	1.16 ± 0.12De
C_2_N_3_	24.24 ± 1.71HIJhi	2.32 ± 0.09HIg	1.11 ± 0.04HIi	0.8 ± 0.04MNn	0.72 ± 0.04IJjk
C_3_N_1_	22.9 ± 1.75IJi	2.22 ± 0.06Igh	1.06 ± 0.06Ii	0.97 ± 0.01KLm	0.91 ± 0.05FGgh
C_3_N_2_	17.34 ± 1.41Kj	1.83 ± 0.04Ji	0.88 ± 0.05Jj	0.68 ± 0.07No	0.77 ± 0.06HIij
C_3_N_3_	15.27 ± 1.98Kj	1.43 ± 0.04Kj	0.63 ± 0.02Kk	0.44 ± 0.05Op	0.7 ± 0.1IJjk
CKJ	46.71 ± 1.22ABab	4.81 ± 0.04Aa	2.75 ± 0.06Bb	3.79 ± 0.07Aa	1.38 ± 0.01ABab
C_1_N_1_J	48.62 ± 0.93Aa	4.84 ± 0.08Aa	2.94 ± 0.08Aa	3.74 ± 0.06Aa	1.27 ± 0.04BCDcd
C_1_N_2_J	38.91 ± 0.54Cd	3.77 ± 0.11Cc	2.65 ± 0.1BCbc	2.72 ± 0.06Dd	1.02 ± 0.06EFf
C_1_N_3_J	27.32 ± 2.12FGg	2.85 ± 0.08Fe	1.61 ± 0.15Fg	1.51 ± 0.06Ij	0.95 ± 0.1FGfgh
C_2_N_1_J	38.83 ± 0.55Cd	3.67 ± 0.17CDc	2.25 ± 0.09De	2.57 ± 0.06Ee	1.15 ± 0.03DEe
C_2_N_2_J	36.14 ± 0.73CDe	2.82 ± 0.07Fe	1.82 ± 0.06Ef	2.12 ± 0.03Gh	1.17 ± 0.06De
C_2_N_3_J	27.82 ± 2.01Fg	2.63 ± 0.16Gf	1.57 ± 0.08Fg	0.99 ± 0.05Klm	0.64 ± 0.01IJk
C_3_N_1_J	26.0 ± 0.77FGHgh	2.51 ± 0.04GHf	1.55 ± 0.08Fg	1.18 ± 0.02Jk	0.77 ± 0.05HIij
C_3_N_2_J	25.5 ± 1.18FGHgh	2.13 ± 0.1Ih	1.36 ± 0.08Gh	0.85 ± 0.02LMn	0.62 ± 0.05Jk
C_3_N_3_J	22.05 ± 2.14Ji	1.9 ± 0.11i	1.04 ± 0.04IJi	0.69 ± 0.05No	0.66 ± 0.66IJk

The data in the table are all moderately different; Different lowercase difference (*P* < 0.05). Different uppercase letters in the same column indicate difference (*P* < 0.01).

#### 3.3.2 Physiological indicators

**Figure 8:**
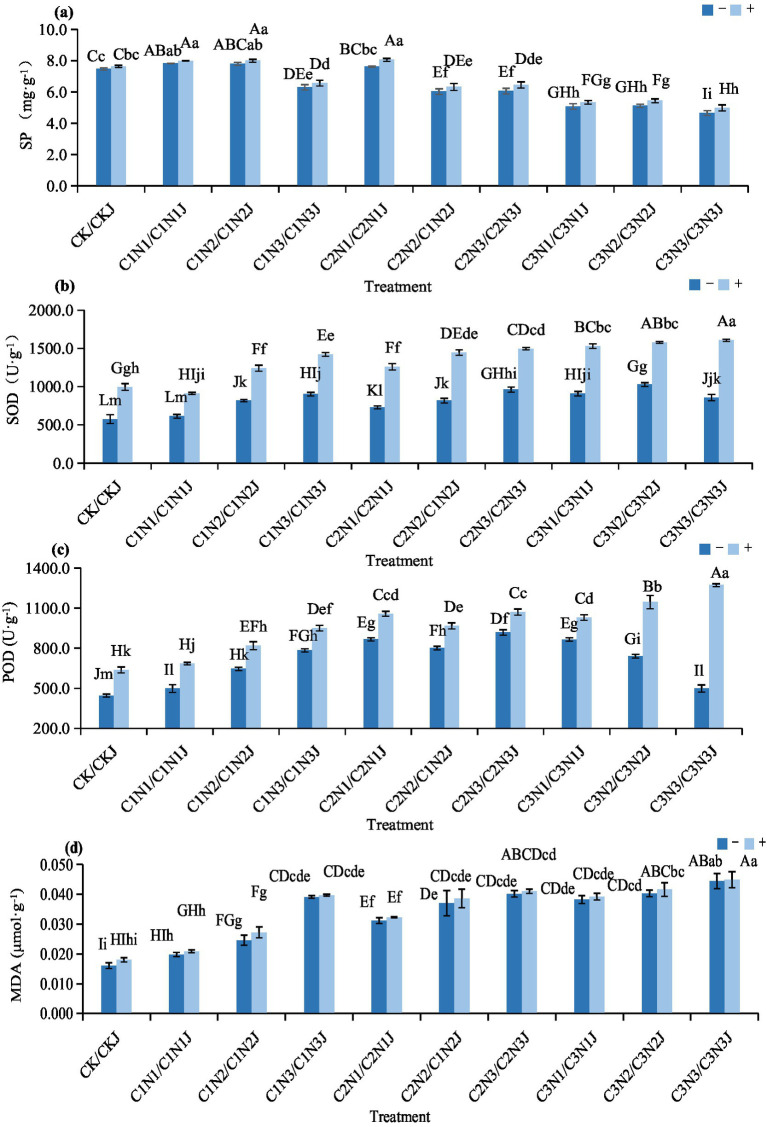
Effects of cadmium and nickel combined stress on physiological indicators of *Z. elegans* mature plants added with *M. algeriense* C14. **(a)** Soluble protein; **(b)** superoxide dismutase; **(c)** peroxidase; **(d)** malondialdehyde. In the figure, different lowercase letters in the same figure indicate significant differences (*p* < 0.05), while different uppercase letters in the same figure indicate extremely significant differences (*p* < 0.01). “−” indicates no application, “+” indicates application.

Under Cd-Ni co-stress, the soluble protein (SP) content, superoxide dismutase (SOD) activity, peroxidase (POD) activity and malondialdehyde (MDA) content of mature *Z. elegans* plants all showed specific change trends with the increase of stress concentration. Inoculation with strain C14 could significantly regulate these physiological indicators to enhance the stress resistance of plants (Figure 8): without the inoculation of the strain, the SP content showed a trend of first increasing and then decreasing with the increase of Cd or Ni concentration. After inoculation with the strain, although it still decreased with the increase of Cd and Ni concentrations, the SP content under the same stress concentration was significantly increased, with the SP content in the C3N3J treatment reaching 4.98 mg·g^−1^, which was higher than 4.65 mg·g^−1^ in the non-inoculated group; the activities of SOD and POD showed an overall upward trend with the increase of stress concentration without the inoculation of the strain, with the SOD activity in the C3N2 treatment reaching 1028.3 U·g^−1^ and the POD activity in the C3N3 treatment reaching 950 U·g^−1^. After inoculation with the strain, both activities were further significantly increased, with the SOD and POD activities in the C3N3J treatment increasing to 1606.92 U·g^−1^ and 1,272 U·g^−1^, respectively; the MDA content increased continuously with the increase of stress concentration, reaching 0.0444 μmol·g^−1^ in the C3N3 treatment without the inoculation of the strain. After inoculation with the strain, the MDA content accumulation under the same concentration of stress was slightly higher than that in the non-inoculated group with no significant difference, with the MDA content in the C3N3J treatment being 0.0449 μmol·g^−1^. The results indicated that strain C14 and enhanced the physiological tolerance of plants to heavy metal stress by increasing the content of osmotic adjustment substances and the activity of antioxidant enzymes.

#### 3.3.3 Enrichment indicators

**Figure 9:**
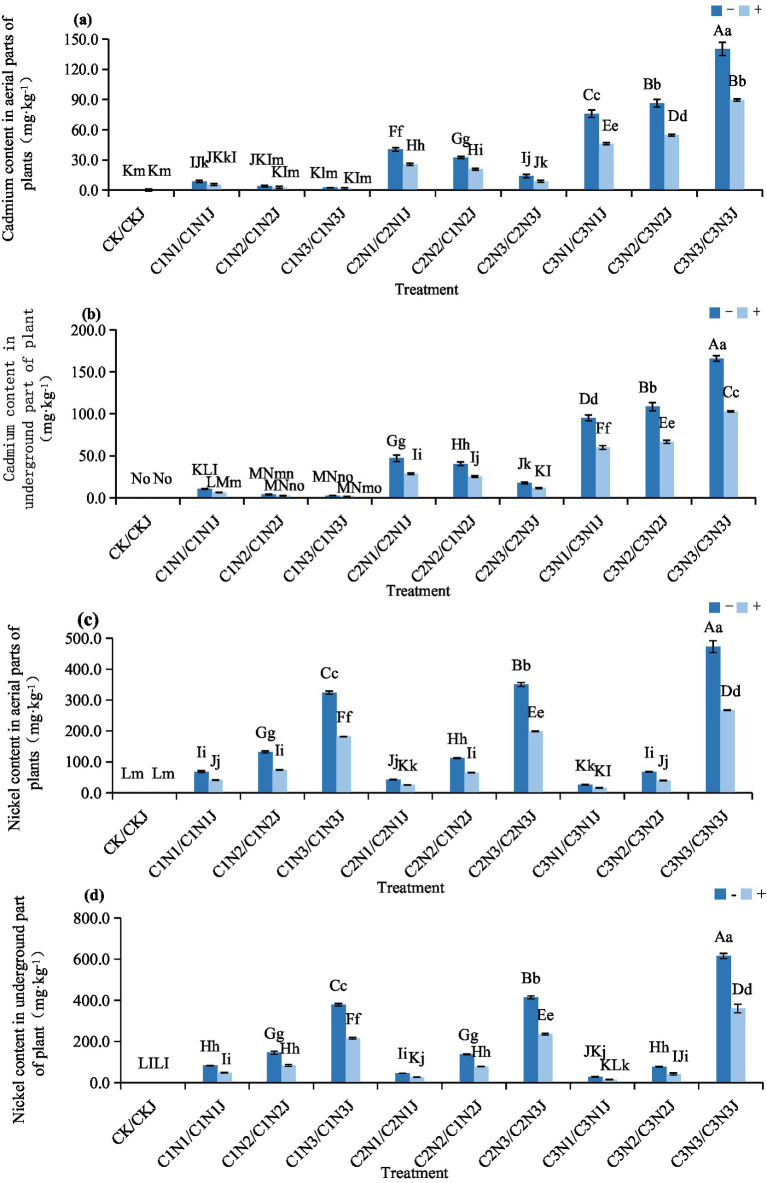
Effects of adding *M. algeriense* C14 on cadmium (**a**: above ground, **b**: below ground) and nickel content (**c**: above ground, **d**: below ground) in *Z. elegans* mature plants under combined cadmium and nickel stress. In the figure, different lowercase letters in the same figure indicate significant differences (*p* < 0.05), and different uppercase letters indicate extremely significant differences (*p* < 0.01); “−” indicates no application, “+” indicates application.

As shown in Figure 9, under Cd-Ni co-stress, the Cd and Ni contents in the aboveground and underground parts of mature *Z. elegans* plants increased significantly with the increase of stress concentration, and the content in the underground parts was always higher than that in the aboveground parts. Without the inoculation of the strain, the Cd and Ni contents in the aboveground parts in the C3N3 treatment (120 mg·L^−1^ Cd^2+^ + 600 mg·L^−1^ Ni^2+^) reached 140.3 mg·kg^−1^ and 473.19 mg·kg^−1^, respectively, and those in the underground parts reached 165.9 mg·kg^−1^ and 615.18 mg·kg^−1^, respectively; under low-concentration Cd stress, Ni would inhibit the Cd uptake by mature plants, while under high-concentration Cd stress, Ni turned into a promoting effect. When the Ni concentration was low, Cd would inhibit the Ni uptake by mature plants, while under high Ni concentration, high Cd concentration promoted Ni accumulation instead. Moreover, the promoting effect of Cd concentration on the Cd content and the inhibitory effect on the Ni content of mature plants were dominant. After inoculation with strain C14, the Cd and Ni contents in the aboveground and underground parts of mature plants under the same concentration of co-stress were significantly reduced, with a decrease range of 15.3–32.7%. The Cd and Ni contents in the aboveground parts in the C3N3J treatment decreased to 89.41 mg·kg^−1^ and 267.05 mg·kg^−1^, respectively, and those in the underground parts decreased to 89.41 mg·kg^−1^ and 360.32 mg·kg^−1^, respectively. At the same time, the inhibitory effect of Cd on Ni uptake was enhanced at each Ni concentration gradient, which effectively reduced the heavy metal accumulation in plants and the risk of heavy metal toxicity.

### 3.4 Correlation analysis

**Figure 10:**
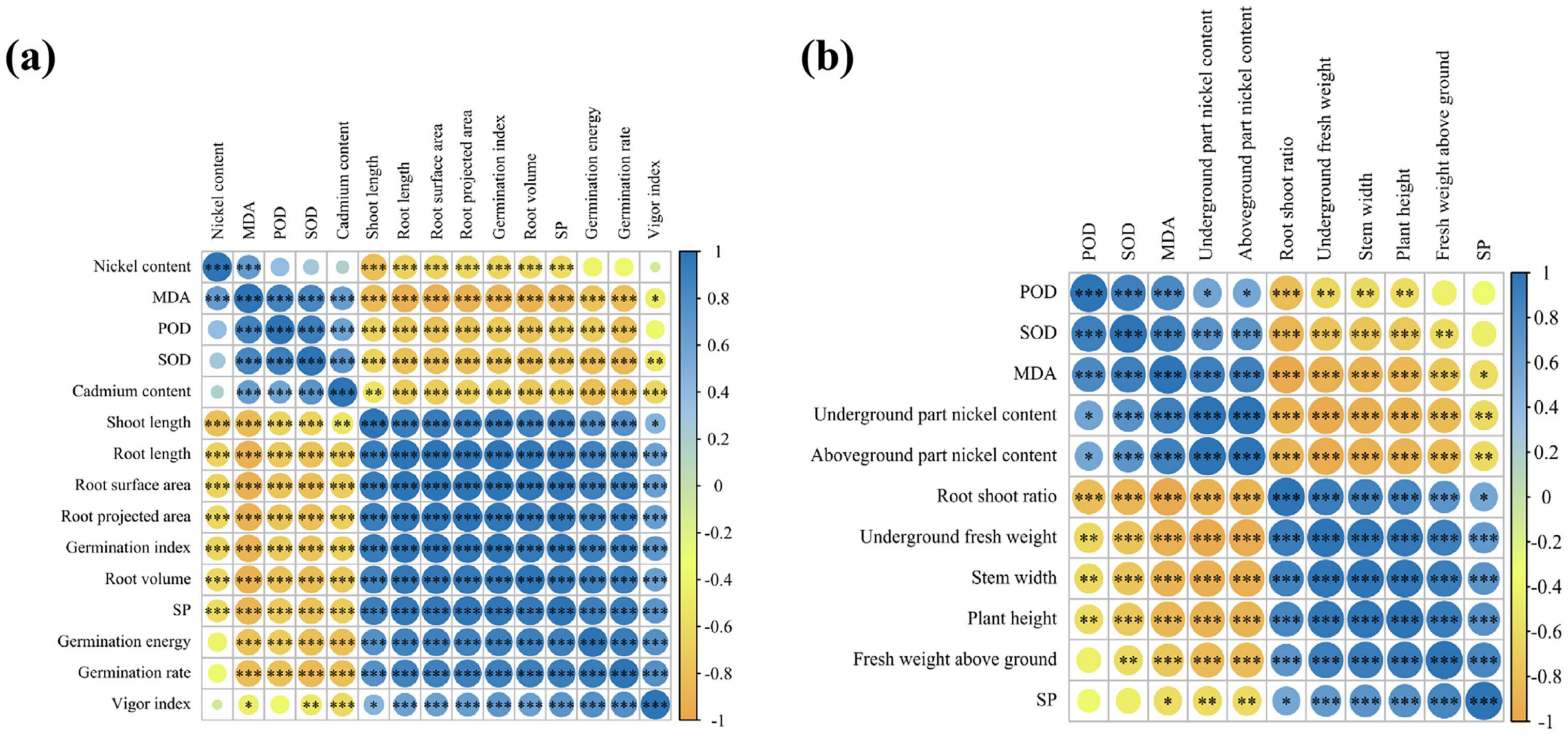
Correlation analysis of the effects of strain C14 inoculation on *Z. elegans* seedlings, mature plant indicators under combined Cd-Ni stress: **(a)** Seedling indicators and **(b)** mature plant indicators.

Under Cd-Ni co-stress, there were significant intrinsic correlations among the growth indicators, physiological indicators and heavy metal enrichment indicators of *Z. elegans* seedlings and mature plants after inoculation with *M. algeriense* C14. After Spearman correlation analysis and heatmap visualization, the index clustering and correlation rules were as follows (Figure 10).

At the seedling stage, the indicators under Cd-Ni co-stress could be clearly clustered into two categories with extremely significant negative correlation (*p* < 0.01): SOD, POD, MDA and plant Cd and Ni accumulation were clustered into the first category, and all indicators in this category were extremely significantly positively correlated (*p* < 0.01), indicating that under Cd-Ni co-stress, the heavy metal accumulation in seedlings increased with the increase of stress degree, which further induced the increase of antioxidant enzyme activities to resist oxidative damage, and at the same time, the degree of membrane lipid peroxidation aggravated and the MDA accumulation increased synchronously; seed germination indicators (germination potential, germination rate, germination index, vigor index), seedling morphological indicators (stem length, root length, root projected area, root surface area, root volume) and SP content were clustered into the second category, and all indicators in this category were also extremely significantly positively correlated (*p* < 0.01), reflecting the coordination between seedling germination potential, morphological formation and osmotic adjustment substance accumulation under co-stress. Among them, the vigor index was significantly positively correlated with germination-related indicators and POD activity, which could comprehensively reflect the germination ability and stress resistance of seedlings under Cd-Ni co-stress.

At the mature plant stage, the index clustering under Cd-Ni co-stress showed obvious differentiation, forming three categories with regular negative correlations: the first category included SOD, POD and Cd contents in aboveground/underground parts of plants; the second category included MDA and Ni contents in aboveground/underground parts of plants. Each category was extremely significantly positively correlated with the corresponding heavy metal content (*p* < 0.01), reflecting that under Cd-Ni co-stress, Cd mainly induced the increase of antioxidant enzyme activities in mature plants, while Ni mainly aggravated the membrane lipid peroxidation damage of mature plants, and the physiological damage targets of the two to mature plants were significantly different; the third category included growth indicators of mature plants (plant height, stem width, aboveground fresh weight, underground fresh weight, root-shoot ratio) and SP content, and the indicators were extremely significantly positively correlated (*p* < 0.01), and this category of indicators was extremely significantly negatively correlated with the first two categories of indicators (*p* < 0.01), indicating that the synergistic toxic effect of Cd and Ni would inhibit the growth and development of mature plants from multiple aspects such as oxidative damage and osmotic regulation. After inoculation with strain C14, the core correlation rules among various indicators did not change, only the correlation coefficients were adjusted, indicating that the strain mainly alleviated the damage of Cd-Ni co-stress by regulating the expression level of each indicator, rather than changing the intrinsic correlation mechanism among indicators.

### 3.5 Principal component analysis

**Figure 11:**
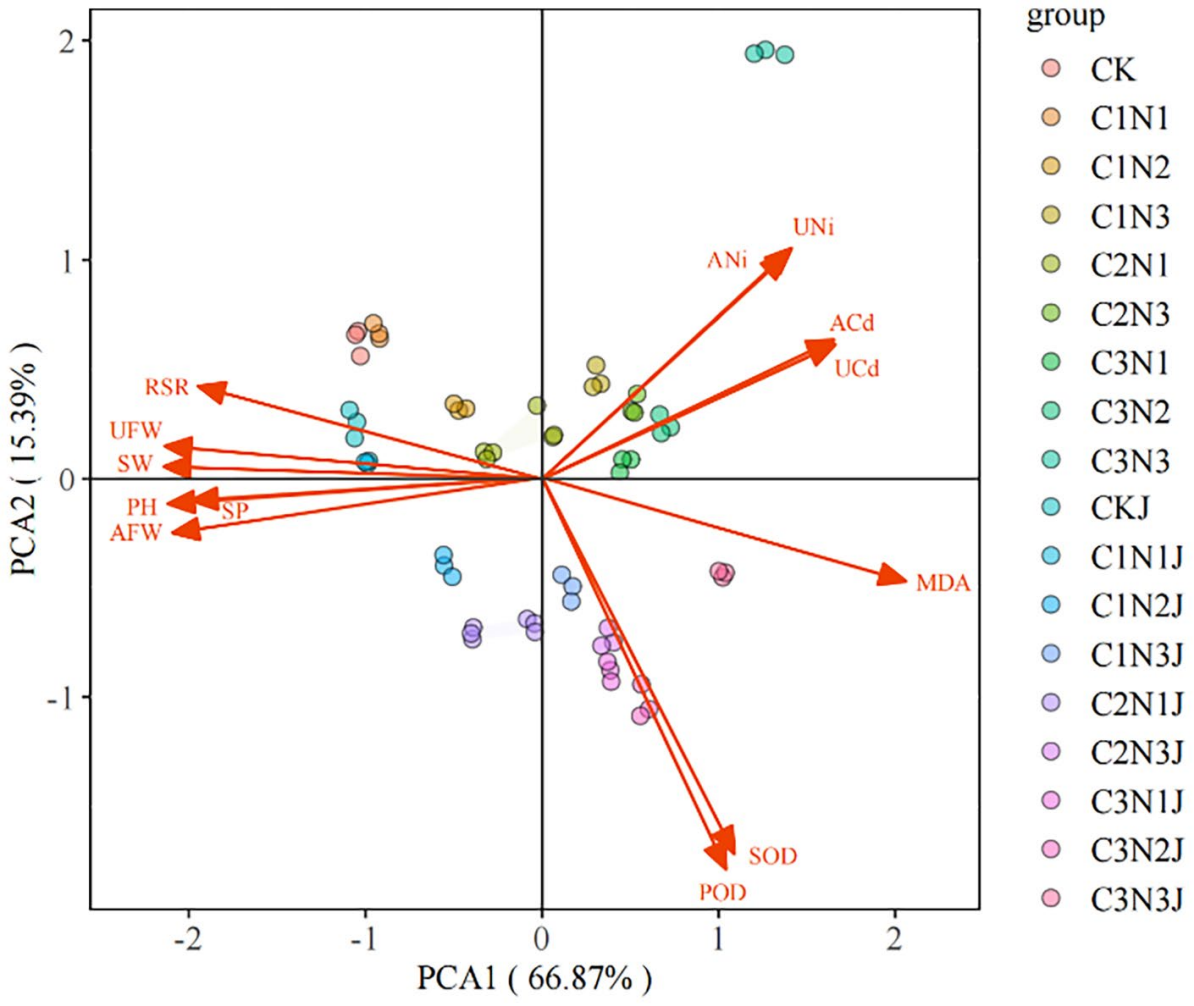
PCA score plot of growth, physiological, and heavy metal indicators of mature *Z. elegans* under Cd-Ni co-stress.

**Figure 12:**
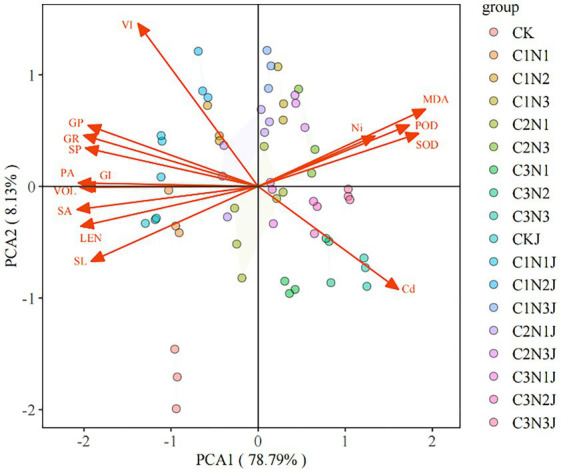
PCA score plot of germination, growth, physiological, and heavy metal indicators of *Z. elegans* seedlings under Cd-Ni co-stress.

To investigate the relationships among crop indicators under the seedling and mature plant conditions, all variables were first standardized. Principal component analysis (PCA) was then performed using the vegan package in R version 4.4.0, and the results were visualized with ggplot2 (as shown in Figures 11, 12). Under the mature plant condition, the first two principal components (PCs) explained 82.26% of the total variance, while under the seedling condition they explained 86.92% of the variance. These results indicate that the first two PCA axes captured the major variation patterns in the data and effectively reduced dimensionality.

Under the mature plant condition, the treatment groups CK, C1N1, CKJ, C1N1J, and C1N2J were distributed on the negative side of the first axis. Along this direction, the indicators Root shoot ratio, Underground fresh weight, Stem width, Plant height, Soluble protein (SP), and Fresh weight above ground clustered together, suggesting that these treatments were strongly influenced by these variables. The groups C1N3J, C2N1J, C2N3J, C3N1J, C3N2J, and C3N3J were located in the fourth quadrant, coinciding with the distribution of Peroxidase (POD) and Superoxide dismutase (SOD), indicating that these treatments were mainly affected by POD and SOD. The groups C1N2, C1N3, C2N1, C2N3, C3N1, C3N2, and C3N3 were situated in the first quadrant and were closely associated with Nickel content of the aboveground part of the plant, Nickel content of the underground part of the plant, Cd content of the aboveground part, Cd content of the underground part, and Malondialdehyde (MDA). These results demonstrated that inoculation with strain C14 altered the overall distribution pattern of treatments, separating inoculated groups from uninoculated ones, and confirmed that strain C14 drove the coordinated regulation of growth, physiological metabolism, and heavy metal uptake in *Z. elegans* under Cd–Ni co-stress.

Under the seedling condition, the CK group was located alone in the third quadrant (negative direction of the second axis) and was strongly influenced by Stem length (SL) and Cd. The groups C1N1, C1N2, CKJ, C1N1J, C1N2J, and C1N3J were distributed on the negative side of the first axis, where Vitality index (VI), Germination potential (GP), Germination rate (GR), Soluble protein (SP), Projected area (PA), Germination index (GI), Volume (VOL), Surface area (SA), Length (LEN), and Stem length (SL) clustered together, indicating that these treatments were mainly affected by these germination and growth indicators. The groups C2N1J, C2N3J, C3N1J, C3N2J, and C3N3J were located in the fourth quadrant, which was dominated by Cd, suggesting a strong influence of Cd on these treatments. The groups C1N2, C1N3, C2N1, C2N3, C3N1, C3N2, and C3N3 were positioned in the first quadrant, where Ni, MDA, POD, and SOD clustered together, indicating that these treatments were primarily influenced by Ni, MDA, POD, and SOD. These results further verified that inoculation with strain C14 significantly changed the multivariate distribution pattern and effectively alleviated Cd–Ni co-stress by regulating growth and physiological performance of *Z. elegans* seedlings.

## 4 Discussion

*Microbacterium algeriense* C14 isolated from the rhizosphere soil of *Z. elegans* in the Cd-Ni contaminated area has strong Cd-Ni resistance, IAA production capacity and heavy metal mineralization capacity, which collectively distinguish it from conventional PGPR that often focus on single metal tolerance or general growth promotion. Mechanistically, strain C14 forms stable carboxylate precipitates through coordination between carboxyl groups in amino acid metabolites and heavy metal ions, thus reducing the bioavailability of heavy metals (Ahemad, 2019; Agarwal et al., 2024; Shehzad et al., 2023; Kumar et al., 2023). This mineralization mechanism is different from the traditional microbially induced carbonate or phosphate precipitation, providing a new approach for heavy metal pollution remediation (Chen et al., 2021; Liu et al., 2025; Ojha et al., 2025; Li et al., 2025).

Heavy metal stress can damage plant cell structure, inhibit photosynthesis and material metabolism, leading to decreased seed germination rate and growth retardation (Ghori et al., 2019; Emamverdian et al., 2015; Hafeez et al., 2023; Rai et al., 2016; Shah et al., 2010). In this study, low concentrations of Cd and Ni had a certain promoting effect on the seed germination of *Z. elegans*, which may be due to low-concentration heavy metals stimulating plants to produce stress responses and activating related metabolic pathways; high concentrations inhibited germination and growth by damaging cell membrane integrity and inhibiting enzyme activity. Inoculation with strain C14 significantly improved seed germination and growth through two key mechanisms: IAA-induced cell elongation and carboxylate-mediated heavy metal immobilization. On the one hand, it benefited from the IAA produced by the strain promoting cell division and elongation; on the other hand, the mineralization of the strain reduced the content of available heavy metals in the soil, alleviating the toxicity of heavy metals to plants.

Plants will produce a large amount of reactive oxygen species under heavy metal stress, leading to lipid peroxidation (MDA accumulation). SOD and POD, as key enzymes in the antioxidant system, can scavenge reactive oxygen species and protect cells from damage (Sharma et al., 2012; Zaid and Wani, 2019; Wang et al., 2019; Nawaz et al., 2021; Sahu et al., 2022). In this study, inoculation with strain C14 significantly increased the SOD and POD activities and soluble protein content of *Z. elegans* under Cd-Ni stress, maintained MDA content at a similar level. This indicates that strain C14 enhances stress tolerance by mechanistically regulating the antioxidant system and osmotic balance to reduce oxidative damage. It indicates that the strain can alleviate the oxidative damage of heavy metal stress to plants by activating the plant antioxidant system and enhancing the osmotic adjustment ability, which is consistent with the physiological regulation effect of PGPR on plants under heavy metal stress in existing studies.

The inhibitory effect of combined Cd-Ni stress on *Z. elegans* was greater than that of single stress, which was due to the synergistic effect of heavy metals aggravating the toxicity to plants. After inoculation with strain C14, the growth, physiology, and rhizosphere environment indicators of *Z. elegans* under combined stress were significantly improved, indicating that the strain also had a mitigation effect on combined heavy metal stress. This mitigation arises from the synergistic mechanism of microbial heavy metal immobilization and plant physiological regulation. However, its effect was affected by the stress concentration. The mitigation effect was weakened under high-concentration combined stress, which may be due to the saturation of the mineralization and growth-promoting capacity of the strain.

## 5 Conclusion

*Microbacterium algeriense* C14 possesses strong Cd–Ni co-resistance, IAA production, and unique carboxylate-mediated mineralization capability that sets it apart from previously reported PGPR. This carboxylate precipitation mechanism differs fundamentally from known immobilization pathways and enables stable long-term reduction of heavy metal bioavailability. The strain can significantly promote the seed germination and growth of *Z. elegans* under single and combined Cd-Ni stress, activate the plant antioxidant system, and reduce the uptake and enrichment of heavy metals; at the same time, it improves the physicochemical properties of the rhizosphere soil, regulates soil enzyme activities and microbial community structure, and alleviates the toxicity of heavy metals to plants. The results show that *M. algeriense* C14 is a high-quality strain for the microbe-plant synergistic remediation of Cd-Ni contaminated soils, providing important theoretical basis and technical support for the ecological remediation of heavy metal-contaminated soils. This study was conducted under controlled laboratory and pot conditions. Due to the complexity of actual contaminated soil environments, the applicability of the results to field-scale remediation needs to be further verified. In the future, field experiments should be conducted to validate the remediation performance and stability of the *M. algeriense* C14–*Z. elegans* system in real heavy metal-contaminated sites, so as to support its practical engineering application.

## 6 Practical relevance and future scope

This study establishes a plant–microbe combined remediation system composed of *Microbacterium algeriense* C14 and *Z. elegans*, which shows high practical value for the *in situ* remediation of cadmium–nickel co-contaminated soil. As a widely cultivated ornamental plant with large biomass and strong adaptability, *Z. elegans* supports both ecological restoration and landscape improvement, avoiding the economic limitations of conventional hyperaccumulators. Strain C14 stabilizes heavy metals through a carboxylate-mediated mineralization mechanism with excellent long-term stability and low risk of metal re-mobilization, making it suitable for the safe utilization of lightly and moderately contaminated farmland and industrial abandoned land.

Future research should focus on field verification in real contaminated sites to evaluate the stability and remediation efficiency of the C14–*Z. elegans* system under complex environmental conditions, explore the combined application of strain C14 with organic amendments or biochar to further strengthen heavy metal immobilization and plant growth promotion, clarify the molecular regulatory network of strain C14 under Cd–Ni stress and its interaction mechanism with the *Z. elegans* rhizosphere, and develop low-cost microbial inoculants suitable for large-scale field application to support engineering popularization and practical application.

## Data Availability

The original contributions presented in the study are included in the article/Supplementary material, further inquiries can be directed to the corresponding authors.
