# Supplementary material for: Effects of Microbacterium algeriense C14 on growth and rhizosphere environment of Zinnia elegans under cadmium and nickel stress
Source: Front Microbiol. 2026 Jun 4;17:1831418. doi: 10.3389/fmicb.2026.1831418 (PMC13275413; doi:10.3389/fmicb.2026.1831418)
Supplement: Supplementary file 1 [file Data_Sheet_1.pdf]

奥维森微生物菌种鉴定

客户姓名	客户单位
刘翰升	吉林农大

样品编号	样品类型	鉴定类型
A-1	组织	16S 鉴定

仪器名称	厂家	型号
测序仪	Applied Biosystems	3730XL
PCR 仪	Applied Biosystems	2720 thermal cycler
板式离心机	Eppendorf	5810R
凝胶成像装置	君意	JY04S-3C
电泳仪	君意	JY300C Power Supply

实验过程：

一. PCR 扩增

以16S通用引物，使用2 ×TsingKE Master Mix（Code No.:TSE003） 体系进行 PCR 扩增，同时以 ddH2O 做负对照，取 2 μl 进行 1%琼脂糖凝胶电泳。

反应体系：

DNA	1 μl
2 ×TsingKE Master Mix	25 μl
F Primer (10 μM)	1 μl
R Primer (10 μM)	1μl
ddH_2O	22 μl
Total	50 μl

反应条件：

94℃	10 min	1 cycle
94℃	30 sec	30 cycles
55℃	30 sec	
72℃	1.5 min	
72℃	10 min	1 cycle

二、取 2 μl 进行 1%琼脂糖凝胶电泳，扩增结果如下：

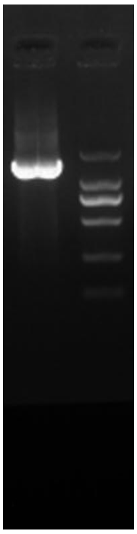

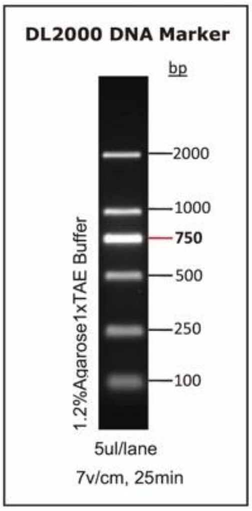

三. PCR 产物纯化

1. 将核对后的 PCR 样品配平离心至 4000 rpm，检查各样品的体积，补水至 50μl
2. 按照样品：6×Loading buffer=5:1 加入 10μl6× Loading buffer，若样品体积在 50-70μl 补加 6×Loading buffer 5μl，离心至 4000 rpm 混匀
3. 将样品点入事先准备好的 1.2%纯化胶中，点样顺序为：纯化胶第一行 1-8 孔点入样品第一列， 第 9 孔为 marker，10-17 孔点入样品第二列。以此类推点完 96孔，最后在纯化胶每行第 9 孔中分别点入 2μl DL2000，在模具侧边贴上所点样品板的日期和板号，例如：0826-E001（标 记是否加急），电泳仪电压设定 160V 接上接头正负极恒压电泳 40-60 min，开始电泳后要观 察纯化胶槽正负极有气泡冒出后，定时。

注意：若样品中有矿物油或者甘油等粘稠物质，可以只吸取下层样品加入，加入时缓慢加入，以 免冲出胶孔，造成样品损失。

4. 将胶块放入凝胶成像仪器中采集图像，图像必须保证 marker 条带清晰。按照日期+板号+第 几次电泳的形式命名胶图，保存在：服务器/测序相关/模板组/胶图/当月的 PCR 电泳记录文 件夹中，如 201408026-E001-S-1/20140826-E001-X-1
5. 对照胶图填写切胶记录预检信息，预检信息用黑色签字笔标记，溶度正常标记√，溶度低标 记半对钩，对于片段大小>1000bp 且总 DNA 量≤300ng 的样品用红色 marker 笔将预检栏圈上标记。
6. 在紫外透射仪下，用手术刀切下目的条带，切取的胶块质量应小于 3g，将其放入对应的板孔号中
7. 4000rpm 离心 1min,加入 500μl Buffer GL，盖上封口膜，65℃水浴 12min，定时

8. 检查每孔胶块是否完全溶解，若没有完全溶解再次 65℃水浴 3min，揭开封口膜，用连续加 液器每孔加入 100μl 混匀的磁珠，对于预检标记为红色的在补入 100μl 磁珠，盖上硅胶垫， 漩涡震荡 30s，转入水平震荡仪 600-800rpm 震荡 5min。
9. 将 96 孔板卡入磁力架中，磁吸 30s，将磁力架和样品正反轻微颠倒 3 次，再次静置磁吸 1min
10. 弃废液，吸水纸上轻磕，用 50-1200μl 8 道电动移液器向每孔移取 500μl BufferW1,盖上硅 胶垫漩涡震荡 30s，将 96 孔板卡入磁力架中，磁吸 30s，将磁力架和样品正反轻微颠倒 3 次， 再次静置磁吸 1min
11. 弃废液，吸水纸上轻磕，用 50-1200μl 8道电动移液器向每孔移取 500μl Buffer W2 盖上硅 胶垫漩涡震荡 30s，将 96 孔板卡入磁力架中，磁吸 30s，将磁力架和样品正反轻微颠倒 3 次， 再次静置磁吸 1min
12. 弃废液，吸水纸上轻磕，倒离心至 600rpm
13. 取下磁力架，加入 35μl 的 Eluent(已 65℃水浴加热)，盖上封口膜，65℃水浴 5min
14. 离心至 1000rpm,将 96 孔板卡入磁力架中，磁吸 1min
15. 2μl 样品+5μl 1.4X 溴酚蓝混合后点入 0.8%的鉴定胶中，按照 A01-H01 的竖向顺序横向点入， 中间空出 2 孔，分别加入 1μl、2μl 量的 DL2000,300V 电泳 11min
16. 将鉴定胶放入凝胶成像仪中采集图像，图像必须保证 marker 条带清晰。按照日期+板号+第几次电泳的形式命名胶图，保存在：服务器/测序相关/模板组/胶图/当月的 PCR 电泳记录文件夹中，如 20140826-E001-S-2/20140826-E001-X-2
17. 对照纯化前后胶图，根据PCR定量标准在PCR记录表上标注每孔模板浓度并稀释至指定浓度，对回收后电泳无条带的样品按照 4μl样品+5μl 1.4× 溴酚蓝再次电泳鉴定
18. 将稀释后模板水浴5 min，离心至4000rpm，标记 Lims 系统模板状态，确认提交前需要再次核对模板状态，确认无误后将模板转交反应组，若有反应同事上班放置于实验桌面，并填 写模板交接记录。若无反应同事上班将模板放置于反应组4℃冰箱保存，并填写模板交接记 录

四. 测序

BigDye® Terminator v3.1 测序反应及纯化

4.1 反应体系:

PCR 纯化产物	1μl
BigDye® Terminator v3.1	2μl
primer（3.2μM）	1μl
ddH_2O	6μl
总体积	10μl

4.2 测序反应循环条件:

96℃	2 min	
96℃	10 sec	30cylce
50℃	10sec	
60℃	3min	
4℃	hold	

4.3 测序反应纯化:

1) 用 8 道移液器往样品板加入 38μl 一步到位,盖硅胶垫震荡 10s,静置 1min,再次震荡 10s,离心至 1000rpm;
2) 将样品板放入磁力架中，卡好，静置 2min;
3) 取下硅胶垫，倒置样板甩弃废液，吸水纸上轻磕;
4) 加入 100μl Magical Buffer，静置 30 秒，倒置样板甩弃废液，吸水纸上轻磕，550rpm 离心 10s，换上新的吸水纸，再次离心 10s;
5) 将样品板从磁力架上取出，放在 96 孔板板托上，自然晾干 2min，加入 20μl 灭菌高纯水。盖上干 净的硅胶垫震荡 30s，4000rpm 离心 1min
6) 从 LIMS 管理系统导出上机表，上 3730 测序仪。

4.4测序数据收集

使用 3730xl 对数据进行收集

(由于前后 20bp 测序峰图碱基信息不准确,因此将 seq 序列文件头尾部分去除后用于 NCBI 比对分析。):

> A-1

GGTGAACACGGAGCTTGCTCTGTGGGATCAGTGGCGAACGGGTGAGTAACACGTGAGCA
ACCTGCCCCTGACTCTGGGATAAGCGCTGGAAACGGCGTCTAATACTGGATATGTGACGT
GATCGCATGGTCTGCGTCTGGAAAGAATTTCGGTTGGGGATGGGCTCGCGGCCTATCAGC
TTGTTGGTGAGGTAATGGCTCACCAAGGCGTCGACGGGTAGCCGGCCTGAGAGGGTGACC
GGCCACACTGGGACTGAGACACGGCCCAGACTCCTACGGGAGGCAGCAGTGGGGAATAT
TGCACAATGGGCGCAAGCCTGATGCAGCAACGCCGCGTGAGGGATGACGGCCTTCGGGTT
GTAAACCTCTTTTAGCAGGGAAGAAGCGAAAGTGACGGTACCTGCAGAAAAAGCGCCGG
CTAACTACGTGCCAGCAGCCGCGGTAATACGTAGGGCGCAAGCGTTATCCGGAATTATTG
GGCGTAAAGAGCTCGTAGGCGGTTTGTCGCGTCTGCTGTGAAATCCGGAGGCTCAACCTC
CGGCCTGCAGTGGGTACGGGCAGACTAGAGTGCGGTAGGGGAGATTGGAATTCCTGGTGT
AGCGGTGGAATGCGCAGATATCAGGAGGAACACCGATGGCGAAGGCAGATCTCTGGGCC
GTAACTGACGCTGAGGAGCGAAAGGGTGGGGAGCAAACAGGCTTAGATACCCTGGTAGT
CCACCCCGTAAACGTTGGGAACTAGTTGTGGGGTCCATTCCACGGATTCCGTGACGCAGC
TAACGCATTAAGTTCCCCGCCTGGGGAGTACGGCCGCAAGGCTAAAACTCAAAGGAATTG
ACGGGGACCCGCACAAGCGGCGGAGCATGCGGATTAATTCGATGCAACGCGAAGAACCT
TACCAAGGCTTGACATATACGAGAACGGGCCAGAAATGGTCAACTCTTTGGACACTCGTA
AACAGGTGGTGCATGGTTGTCGTCAGCTCGTGTCGTGAGATGTTGGGTTAAGTCCCGCAA
CGAGCGCAACCCTCGTTCTATGTTGCCAGCACGTAATGGTGGGAACTCATGGGATACTGC
CGGGGTCAACTCGGAGGAAGGTGGGGATGACGTCAAATCATCATGCCCCTTATGTCTTGG
GCTTCACGCATGCTACAATGGCCGGTACAAAGGGCTGCAATACCGCGAGGTGGAGCGAA
TCCCAAAAAGCCGGTCCCAGTTCGGATTGAGGTCTGCAACTCGACCTCATGAAGTCGGAG
TCGCTAGTAATCGCAGATCAGCAACGCTGCGGTGAATACGTTCCCGGGTCTTGTACACAC
CGCCCGTCAAGTCATGAAAGTCGGTAACACCTGAAGCCGGTGGCCTAA

鉴定结果

Job Title	Nucleotide Sequence
RID	KY3T2TC1013 *Search expires on 10-31 10:04 am* Download All
Program	BLASTN Citation
Database	rRNA_typestrains/16S_ribosomal_RNA See details
Query ID	lcl|Query_14671
Description	None
Molecule type	dna
Query Length	1361
Other reports	Distance tree of results MSA viewer

Filter Results

Organism *only top 20 will appear* exclude

Type common name, binomial, taxid or group name

+ Add organism

Percent Identity to **E value** to **Query Coverage** to

Filter Reset

Descriptions | Graphic Summary | Alignments | Taxonomy

Sequences producing significant alignments Download Select columns Show 100

select all *100 sequences selected* GenBank Graphics Distance tree of results MSA Viewer

Description	Scientific Name	Max Score	Total Score	Query Cover	E value	Per. Ident	Acc. Len	Accession
Microbacterium algeriense strain G1 16S ribosomal RNA, partial sequence	Microbacterium algeriense	2514	2514	100%	0.0	100.00%	1426	NR_180420.1
Microbacterium oxydans strain DSM 20578 16S ribosomal RNA, partial sequence	Microbacterium oxydans	2499	2499	100%	0.0	99.78%	1466	NR_044931.1
Microbacterium maritypicum strain DSM 12512 16S ribosomal RNA, partial sequence	Microbacterium maritypicum	2490	2490	100%	0.0	99.71%	1437	NR_042351.1
Microbacterium liquefaciens strain DSM 20638 16S ribosomal RNA, partial sequence	Microbacterium liquefaciens	2481	2481	100%	0.0	99.56%	1474	NR_026162.1
Microbacterium luteolum strain DSM 20143 16S ribosomal RNA, partial sequence	Microbacterium luteolum	2470	2470	100%	0.0	99.19%	1462	NR_119269.1
Microbacterium saperdae strain DSM 20169 16S ribosomal RNA, partial sequence	Microbacterium saperdae	2466	2466	100%	0.0	99.19%	1473	NR_119270.1
Microbacterium paraoxydans strain CF36 16S ribosomal RNA, partial sequence	Microbacterium paraoxydans	2464	2464	100%	0.0	99.34%	1490	NR_025548.1
Microbacterium saperdae strain IFO 15038 16S ribosomal RNA, partial sequence	Microbacterium saperdae	2440	2440	100%	0.0	98.97%	1407	NR_024637.1
Microbacterium luteolum strain IFO 15074 16S ribosomal RNA, partial sequence	Microbacterium luteolum	2435	2435	100%	0.0	98.75%	1440	NR_024636.1
Microbacterium hydrocarbonoxydans strain BNP48 16S ribosomal RNA, partial sequence	Microbacterium hydrocarbonoxydans	2425	2425	100%	0.0	98.83%	1495	NR_042263.1
Microbacterium foliorum strain P 333/02 16S ribosomal RNA, partial sequence	Microbacterium foliorum	2414	2414	100%	0.0	98.68%	1480	NR_025368.1
Microbacterium ginsengiterrae strain DCY37 16S ribosomal RNA, partial sequence	Microbacterium ginsengiterrae	2409	2409	100%	0.0	98.61%	1427	NR_116483.1
Microbacterium phyllosphaerae strain P 369/06 16S ribosomal RNA, partial sequence	Microbacterium phyllosphaerae	2405	2405	99%	0.0	98.82%	1478	NR_025405.1
Microbacterium maritypicum strain DSM 12512 16S ribosomal RNA, partial sequence	Microbacterium maritypicum	2392	2392	95%	0.0	99.85%	1345	NR_114986.1

此鉴定结果仅供科研。如对鉴定结果有异议，请于一个月内提出。
