# Supplementary material for: Effects of Microbacterium algeriense C14 on growth and rhizosphere environment of Zinnia elegans under cadmium and nickel stress
Source: Front Microbiol. 2026 Jun 4;17:1831418. doi: 10.3389/fmicb.2026.1831418 (PMC13275413; doi:10.3389/fmicb.2026.1831418)
Supplement: Supplementary file 2 [file Data_Sheet_2.pdf]

Identification of Microbial Strains by Ovison

Customer Name	Customer unit
Liu Hansheng	Jilin Agricultural University

Sample number	Sample type	Identification type
A-1	Organization	16S identification

Instrument name	Vender	Model
Sequenator	Applied Biosystems	3730XL
PCR instrument	Applied Biosystems	2720 thermal cycler
Plate-type centrifuge	Eppendorf	5810R
Gel imaging device	Your Majesty	JY04S-3C
Electrophoresis apparatus trophoresis	Your Majesty	JY300C Power Supply

experimentation：

I. PCR amplification

PCR amplification was performed using universal 16S rRNA primers with the 2×TsingKE Master Mix system (Code No.: TSE003), with ddH2O as negative control. A sample of 2 µl was subjected to 1% agarose gel electrophoresis.

reaction system：

DNA	1 µl
2 ×TsingKE Master Mix	25 µl
F Primer (10 µM)	1 µl
R Primer (10 µM)	1 µl
ddH_2O	22 µl
Total	50 µl

reaction condition：

94℃	10 min	1 cycle
94℃	30 sec	30 cycles
55℃	30 sec	
72℃	1.5 min	
72℃	10 min	1 cycle

2. Take 2 µl for 1% agarose gel electrophoresis, with the amplification results as follows:

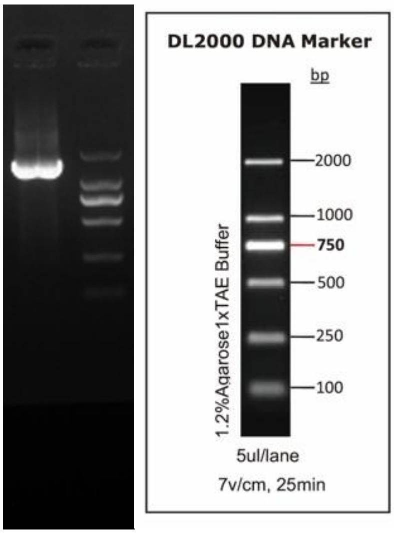

III. Purification of PCR Products

1. Centrifuge the verified PCR samples at 4000 rpm, check the volume of each sample, and add water to a final volume of 50 µl.
2. Add 10 µl of 6× Loading buffer according to the sample: 6× Loading buffer = 5:1. If the sample volume is 50–70 µl, add 5 µl of 6× Loading buffer. Centrifuge at 4000 rpm to mix thoroughly.
3. Apply samples to the pre-prepared 1.2% purification gel in the following sequence: For the first row of the gel, place samples in wells 1-8 (first column), use well 9 as a marker, and fill wells 10-17 with samples from the second column. Repeat this pattern across all 96 wells. Finally, add 2 µl of DL2000 to each well in the 9th position of every row. Label the sample plate with its date and plate number (e.g., 0826-E001) and indicate whether urgent processing is required. Set the electrophorizer voltage to 160V, connect the adapter, and perform constant-voltage electrophoresis for 40-60 minutes. After initiation, observe for bubble formation at the positive and negative electrodes of the gel tank and record time points accordingly.

pay attention to： If the sample contains viscous substances such as mineral oil or glycerol, only the lower layer of the sample may be aspirated and added. The addition should be performed slowly to avoid flushing out the gel pores, which could lead to sample loss.

4. Place the gel block into the gel imaging instrument for image acquisition, ensuring that the marker bands are clearly visible. Name the gel electrophoresis images using the format of date + plate number + number of electrophoresis runs, and save them in the following directory: server/sequencing-related/template group/gel images/monthly PCR electrophoresis record folder, e.g., 201408026-E001-S-1/20140826-E001-X-1.

5. Fill in the cutting record pre-examination information using the control gel image, and mark the pre-examination information with a black marker pen. Mark with a square (√) for normal solubility and a half-check for low solubility. For samples with fragment size>1000 bp and total DNA content ≤300 ng, use a red marker pen to circle and mark the pre-examination column.

6. Under the ultraviolet transmittance spectrometer, the target strip was excised with a surgical knife. The mass of the excised gel block should be less than 3 g, and it was placed into the corresponding well number on the plate.

7. Centrifuge at 4000 rpm for 1 minute, add 500 µl Buffer GL, cover with sealing film, and incubate in a water bath at 65°C for 12 minutes, with timer set.

8. Check whether the gel blocks in each well have completely dissolved. If not, reheat in a 65°C water bath for 3 minutes, remove the sealing film, and add 100 μl of the mixed magnetic beads per well using a continuous feeder. For wells pre-labeled red, add an additional 100 μl of magnetic beads. Cover with a silica gel pad, vortex震荡 for 30 seconds, then transfer to a horizontal shaker and shake at 600–800 rpm for 5 minutes.
9. Insert the 96-well plate into the magnetic rack, magnetize for 30 seconds, gently invert the magnetic rack and samples three times, then allow another 1-minute static magnetization period.
10. Discard waste liquid, gently tap with absorbent paper, and use a 50-1200 μ l 8-channel electric pipette to transfer 500 μ l BufferW1 into each well. Cover with a silicone pad and vortex for 30 seconds. Insert the 96-well plate into the magnetic rack, magnetize for 30 seconds, then gently invert the magnetic rack and samples three times. Magnetize again for 1 minute.
11. Discard waste liquid, gently tap with absorbent paper, and transfer 500 μ l of Buffer W2 using a 50-1200 μ l eight-channel electric pipette to each well. Cover with a silicone pad and vortex for 30 seconds. Insert the 96-well plate into the magnetic rack, magnetize for 30 seconds, then gently invert the magnetic rack and samples three times. Allow another 1-minute static magnetization period.
12. Discard waste liquid, gently tap with absorbent paper, and centrifuge at 600 rpm
13. Remove the magnetic rack, add 35 μ l of eluent (preheated in a 65°C water bath), cover with a sealing film, and incubate in a 65°C water bath for 5 minutes.
14. Centrifuge at 1000 rpm, then insert the 96-well plate into the magnetic rack and magnetize for 1 minute.
15. Combine 2 μ l of sample with 5 μ l of 1.4X bromophenol blue solution, then spot the mixture onto 0.8% gel for identification. Perform horizontal spotting in the vertical sequence specified in A01-H01, leaving 2 intermediate wells empty. Add 1 μ l and 2 μ l of DL2000 solution respectively, followed by 300 V electrophoresis for 11 minutes.
16. Place the gel to be analyzed into the gel imaging system for image acquisition, ensuring that the marker bands are clearly visible. Name the gel electrophoresis images using the format of date + plate number + number of electrophoresis runs, and save them in the following folder: server/sequencing-related/template group/gel images/ monthly PCR electrophoresis record folder, e.g., 20140826-E001-S-2/20140826-E001-X-2.
17. Compare the gel images before and after purification, and according to PCR quantification standards, record the template concentration per well on the PCR record sheet and dilute to the specified concentration. For samples showing no bands after electrophoresis, perform re-electrophoresis with 4 μ l sample + 5 μ l 1.4× bromophenol blue for identification.
18. Place the diluted template in a water bath for 5 minutes, then centrifuge at 4000 rpm to label the template status in the Lims system. Verify the template status again before submission. After confirmation, transfer the template to the reaction group. If there is no reaction, colleagues should place the template on the laboratory table upon returning to work and complete the template handover record. If there is a reaction, colleagues should store the template in the reaction group's 4°C refrigerator upon returning to work and fill out the template handover record.

IV. Sequencing

BigDye® Terminator v3.1 sequencing reaction and purification

4.1 reaction system：

PCR purified product	1μl
BigDye® Terminator v3.1	2μl
primer（3.2μM）	1μl
ddH_2O	6μl
bulk volume	10μl

4.2 Sequencing reaction cycle conditions:

96℃	2 min	
96℃	10 sec	30cylce
50℃	10sec	
60℃	3min	
4℃	hold	

4.3 Purification of sequencing reactions:

1) Add 38 μl of sample to the sample plate using an 8-port pipette, transfer in one step, cover with a silica gel pad and shake for 10 seconds, let stand for 1 minute, shake again for 10 seconds, then centrifuge at 1000 rpm.
2) Place the sample plate into the magnetic rack, secure it, and allow it to rest for 2 minutes.
3) Remove the silica gel pad, invert the sample plate to discard the waste liquid, and gently tap with absorbent paper.
4) Add 100 μl of Magical Buffer, incubate for 30 seconds, invert the plate to discard waste liquid, gently tap with absorbent paper, centrifuge at 550 rpm for 10 seconds, replace with fresh absorbent paper, and repeat centrifugation for another 10 seconds.
5) Remove the sample plate from the magnetic rack and place it on the 96-well plate holder. Allow natural drying for 2 minutes, then add 20 μl of sterile high-purity water. Cover with a clean silica gel pad and shake for 30 seconds, followed by centrifugation at 4000 rpm for 1 minute.
6) Export the computer log from the LIMS management system to the 3730 sequencer.

4.4 Sequencing Data Collection

Use 3730xl to collect data

Due to inaccurate base information in the 20bp sequencing peak maps before and after the region, the head and tail portions of the seq sequence file were removed prior to NCBI alignment analysis.

> A-1 GGTGAACACGGAGCTTGCTCTGTGGGATCAGTGGCGAACGGGTGAGTAACACGTGA
GCAACCTGCCCCTGACTCTGGGATAAGCGCTGGAAACGGCGTCTAATACTGGATATGTGA
CGT GATCGCATGGTCTGCGTCTGGAAAGAATTTCGGTTGGGGATGGGCTCGCGGCCTATC
AGC TTGTTGGTGAGGTAATGGCTCACCAAGGCGTCGACGGGTAGCCGGCCTGAGAGGGTG
ACC GGCCACACTGGGACTGAGACACGGCCCAGACTCCTACGGGAGGCAGCAGTGGGGAA
TAT TGCACAATGGGCGCAAGCCTGATGCAGCAACGCCGCGTGAGGGATGACGGCCTTCGG
GTT GTAAACCTCTTTTAGCAGGGAAGAAGCGAAAGTGACGGTACCTGCAGAAAAAGCGC
CGGCTAACTACGTGCCAGCAGCCGCGGTAATACGTAGGGCGCAAGCGTTATCCGGAATTA
TTGGGCGTAAAGAGCTCGTAGGCGGTTTGTCGCGTCTGCTGTGAAATCCGGAGGCTCAACCTC
CGGCCTGCAGTGGGTACGGGCAGACTAGAGTGCGGTAGGGGAGATTGGAATTCCTGGTGT
AGCGGTGGAATGCGCAGATATCAGGAGGAACACCGATGGCGAAGGCAGATCTCTGGGCC
GTAACTGACGCTGAGGAGCGAAAGGGTGGGGAGCAAACAGGCTTAGATACCCTGGTAGT
CCACCCCGTAAACGTTGGGAACTAGTTGTGGGGTCCATTCCACGGATTCCGTGACGCAGC
TAACGCATTAAGTTCCCCGCCTGGGGAGTACGGCCGCAAGGCTAAAACTCAAAGGAATTG
ACGGGGACCCGCACAAGCGGCGGAGCATGCGGATTAATTCGATGCAACGCGAAGAACCT
TACCAAGGCTTGACATATACGAGAACGGGCCAGAAATGGTCAACTCTTTGGACACTCGTA
AACAGGTGGTGCATGGTTGTCGTCAGCTCGTGTCGTGAGATGTTGGGTTAAGTCCCGCAA
CGAGCGCAACCCTCGTTCTATGTTGCCAGCACGTAATGGTGGGAACTCATGGGATACTGC
CGGGGTCAACTCGGAGGAAGGTGGGGATGACGTCAAATCATCATGCCCCTTATGTCTTGG
GCTTCACGCATGCTACAATGGCCGGTACAAAGGGCTGCAATACCGCGAGGTGGAGCGAA
TCCCAAAAAGCCGGTCCCAGTTCGGATTGAGGTCTGCAACTCGACCTCATGAAGTCGGAG
TCGCTAGTAATCGCAGATCAGCAACGCTGCGGTGAATACGTTCCCGGGTCTTGTACACAC
CGCCCGTCAAGTCATGAAAGTCGGTAACACCTGAAGCCGGTGGCCTAA

Identification Result

Job Title	**Nucleotide Sequence**
RID	KY3T2TC1013 *Search expires on 10-31 10:04 am* Download All
Program	BLASTN Citation
Database	rRNA_typestrains/16S_ribosomal_RNA See details
Query ID	lcl|Query_14671
Description	None
Molecule type	dna
Query Length	1361
Other reports	Distance tree of results MSA viewer

Filter Results

Organism *only top 20 will appear* exclude

Type common name, binomial, taxid or group name

+ Add organism

Percent Identity to **E value** to **Query Coverage** to

Filter Reset

Descriptions | Graphic Summary | Alignments | Taxonomy

Sequences producing significant alignments Download Select columns Show 100

select all *100 sequences selected* GenBank Graphics Distance tree of results MSA Viewer

Description	Scientific Name	Max Score	Total Score	Query Cover	E value	Per. Ident	Acc. Len	Accession
Microbacterium algeriense strain G1 16S ribosomal RNA, partial sequence	Microbacterium algeriense	2514	2514	100%	0.0	100.00%	1426	NR_180420.1
Microbacterium oxydans strain DSM 20578 16S ribosomal RNA, partial sequence	Microbacterium oxydans	2499	2499	100%	0.0	99.78%	1466	NR_044931.1
Microbacterium maritypicum strain DSM 12512 16S ribosomal RNA, partial sequence	Microbacterium maritypicum	2490	2490	100%	0.0	99.71%	1437	NR_042351.1
Microbacterium liquefaciens strain DSM 20638 16S ribosomal RNA, partial sequence	Microbacterium liquefaciens	2481	2481	100%	0.0	99.56%	1474	NR_026162.1
Microbacterium luteolum strain DSM 20143 16S ribosomal RNA, partial sequence	Microbacterium luteolum	2470	2470	100%	0.0	99.19%	1462	NR_119269.1
Microbacterium saperdae strain DSM 20169 16S ribosomal RNA, partial sequence	Microbacterium saperdae	2466	2466	100%	0.0	99.19%	1473	NR_119270.1
Microbacterium paraoxydans strain CF36 16S ribosomal RNA, partial sequence	Microbacterium paraoxydans	2464	2464	100%	0.0	99.34%	1490	NR_025548.1
Microbacterium saperdae strain IFO 15038 16S ribosomal RNA, partial sequence	Microbacterium saperdae	2440	2440	100%	0.0	98.97%	1407	NR_024637.1
Microbacterium luteolum strain IFO 15074 16S ribosomal RNA, partial sequence	Microbacterium luteolum	2435	2435	100%	0.0	98.75%	1440	NR_024636.1
Microbacterium hydrocarbonoxydans strain BNP48 16S ribosomal RNA, partial sequence	Microbacterium hydrocarbonoxydans	2425	2425	100%	0.0	98.83%	1495	NR_042263.1
Microbacterium foliorum strain P 333/02 16S ribosomal RNA, partial sequence	Microbacterium foliorum	2414	2414	100%	0.0	98.68%	1480	NR_025368.1
Microbacterium ginsengiterrae strain DCY37 16S ribosomal RNA, partial sequence	Microbacterium ginsengiterrae	2409	2409	100%	0.0	98.61%	1427	NR_116483.1
Microbacterium phyllosphaerae strain P 369/06 16S ribosomal RNA, partial sequence	Microbacterium phyllosphaerae	2405	2405	99%	0.0	98.82%	1478	NR_025405.1
Microbacterium maritypicum strain DSM 12512 16S ribosomal RNA, partial sequence	Microbacterium maritypicum	2392	2392	95%	0.0	99.85%	1345	NR_114986.1

This identification result is for scientific research purposes only. If you have any objections to the identification result, please raise them within one month.
